# Toward Analysis at the Point of Need: A Digital Microfluidic Approach to Processing Multi‐Source Sexual Assault Samples

**DOI:** 10.1002/advs.202405712

**Published:** 2024-09-04

**Authors:** Mohamed Elsayed, Leticia Bodo, Christine Gaoiran, Palig Keuhnelian, Advikaa Dosajh, Vivienne Luk, Melissa Schwandt, Julie L. French, Alpana Ghosh, Barbara Erickson, Amanda G. Charlesworth, Jonathan Millman, Aaron R. Wheeler

**Affiliations:** ^1^ Institute of Biomedical Engineering University of Toronto 164 College Street Toronto ON M5S 3E2 Canada; ^2^ Donnelly Centre for Cellular and Biomolecular Research University of Toronto 160 College Street Toronto ON M5S 3E1 Canada; ^3^ Department of Chemistry University of Toronto 80 St. George Street Toronto ON M5S 3H6 Canada; ^4^ Forensic Science Department University of Toronto Mississauga 4th floor, Terrence Donnelly Health Sciences Complex, 3359 Mississauga Rd. Mississauga ON L5L 1C6 Canada; ^5^ ANDE Corporation 1860 Industrial Circle, Suite A Longmont CO 80501 USA; ^6^ Centre of Forensic Sciences 25 Morton Shulman Avenue Toronto ON M3M 0B1 Canada

**Keywords:** differential digestion, digital microfluidics, rapid DNA analysis

## Abstract

Forensic case samples collected in sexual assaults typically contain DNA from multiple sources, which complicates short‐tandem repeat (STR) profiling. These samples are typically sent to a laboratory to separate the DNA from sperm and non‐sperm sources prior to analysis. Here, the automation and miniaturization of these steps using digital microfluidics (DMF) is reported, which may eventually enable processing sexual assault samples outside of the laboratory, at the point of need. When applied to vaginal swab samples collected up to 12 h post‐coitus (PC), the new method identifies single‐source (male) STR profiles. When applied to samples collected 24–72 h PC, the method identifies mixed STR profiles, suggesting room for improvement and/or potential for data deconvolution. In sum, an automated, miniaturized sample pre‐processing method for separating the DNA contained in sexual assault samples is demonstrated. This type of automated processing using DMF, especially when combined with Rapid DNA Analysis, has the potential to be used for processing of sexual assault samples in hospitals, police offices, and other locations outside of the laboratory.

## Graphical abstract

An automated, potentially portable Differential Digestion technique is developed using digital microfluidics. The new method proved compatible with Rapid DNA analysis, resulting in high‐quality STR profiles that can be used to identify the male profile in vaginal swab samples collected after coitus.

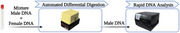

## 1 Introduction

There are almost half a million sexual assaults in Canada each year and over 90% of sexual assault cases go unreported.^[^ ^1^ ^]^ In fact, public perception of the slow analysis of forensic evidence in sexual assault cases drives reluctance among complainants to come forward, and it is widely understood that faster analysis would give victims more confidence to report assaults.^[^ ^2^ ^]^ There is thus great interest in the development of methods for rapid testing of forensic samples in sexual assault cases. To tackle this problem, it is useful to review the steps involved in a sexual assault sample analysis.

In a typical sexual assault, forensic evidence is collected from victims and/or the scenes of crime (the “case” samples). The evidence is processed for biological (DNA) and other items of evidentiary value which can be used to aid the investigation of the criminal offense. Case samples are traditionally 1) transported to a laboratory and 2) entered into a queue prior to analysis. Once they make their way through the queue, if the case samples contain DNA from multiple individuals, they must be 3A) processed to separate the DNA from the different sources. Then, the case samples are 3B) analyzed, which entails extraction of the DNA, cleanup, PCR amplification, and electrophoretic analysis to identify the alleles at the short tandem repeat (STR) loci. The STR profiles of the samples can then be compared to reference samples collected from known individuals and to profiles in national and international databases. Steps 3A‐3B) can be completed in a few hours of laboratory time,^[^ ^3^ ^]^ which represents a small contribution to the whole. The bottleneck in this process is steps 1‐2), which can extend the duration to days, weeks, or even longer.^[^ ^2^, ^3^, ^4^ ^]^

The last decade has seen an important innovation in this space – the development of “Rapid DNA” systems that automate step 3B). That is, Rapid DNA systems allow for automated extraction, purification, amplification, and separation in a sample‐to‐answer format that can be implemented outside of the lab.^[^ ^5^, ^6^ ^]^ For example, the ANDE Rapid DNA System used here 1) uses a pneumatic system to introduce a lysing buffer into the swab chambers, 2) binds the extracted DNA to a silica membrane for DNA purification, 3) elutes the purified DNA, 4) mixes the purified DNA with PCR master mix including the STR primers, 5) amplifies STR markers in the DNA, 6) separates the PCR amplicons using capillary electrophoresis in microchannels, 7) measures fluorescent intensities of the DNA fragments to produce electropherograms for each sample, and 8) interprets the electropherogram using the onboard analysis software. An advantage for Rapid DNA systems is their mobility; they are now being used regularly in settings outside of the laboratory, including police stations, mobile crime scene units, field‐forward military missions, and mass disaster settings.^[^ ^7^, ^8^, ^9^ ^]^ A disadvantage for Rapid DNA systems is a lack of integrated software to deconvolute mixed‐sample data. That is, while Rapid DNA systems (today) are well suited for automatically evaluating samples containing a single individual's DNA, the interpretation of data generated from samples containing a mixture of DNA from more than one individual requires assistance from a trained analyst to apply cutting‐edge data review/software algorithms for deconvolution. This disadvantage is what drives the work presented here – developing means to separate DNA from more than one individual prior to analysis on the Rapid DNA system.

Unfortunately, while Rapid DNA systems form an elegant solution to automating step 3B), there is no such elegant/mobile solution for automating step 3A), which is typically performed in the laboratory. There are a number of methods that have been proposed to address this challenge, including micromanipulation,^[^ ^10^ ^]^ laser‐capture microdissection,^[^ ^11^, ^12^ ^]^ fluorescence activated cell sorting,^[^ ^13^ ^]^ magnetic activated cell sorting,^[^ ^13^, ^14^ ^]^ acoustophoresis,^[^ ^15^, ^16^, ^17^ ^]^ optical tweezers,^[^ ^18^, ^19^ ^]^ and dielectrophoresis.^[^ ^20^ ^]^ There has been exciting innovation in this space, but little has made it into practical use by the forensic community because these methods typically require detailed attention from a trained and experienced operator, in methods that are by nature low throughput (e.g., a single sample can require 24 h of total processing time^[^ ^20^ ^]^), making them ill‐suited for routine use or in mobile applications.

The forensic community has largely opted to address step 3A) using a technique known as differential extraction (DE). DE was introduced in 1985 by Gill et al.^[^ ^21^ ^]^ as a method to extract male DNA from mixed samples. The original technique required overnight lysis steps; in 1995, Yoshida et al.^[^ ^22^ ^]^ developed an improved method to enable the full process to be completed in 8 h and to improve extraction efficiency. Since that time there have been additional improvements in extraction efficiency making use of different lysis conditions,^[^ ^23^, ^24^, ^25^, ^26^ ^]^ as well as the development of related techniques, such as differential digestion (DD).^[^ ^3^ ^]^ Even with these improvements, DE (and DD, and others) is typically a manual process requiring a well‐equipped laboratory and a skilled operator to execute it successfully. For example, given the complexity of DE, in many forensic analysis laboratories, DNA analysts must undergo rigorous training that can take several months to complete before they are allowed to apply this procedure to case samples. Lack of access to adequately trained analysts has been a problem, causing some sexual assault samples not to be tested due to extensive backlogs.^[^ ^2^, ^4^, ^27^, ^28^, ^29^ ^]^ When these problems are addressed by turning to inexperienced analysts, mistakes can be made, which can complicate the results or even invalidate them.^[^ ^30^ ^]^ Finally, even when carried out by experienced technicians, ≈60% of male DNA is lost in a typical DE process,^[^ ^30^, ^31^ ^]^ which can make the subsequent STR analysis more challenging.

Given the bottlenecks and complexities described above, we propose a radical new approach that could streamline the analysis pipeline in sexual assault sample analysis. If step 3A) could be automated and implemented in a format such that it could be used for mobile applications outside of the laboratory, if used with Rapid DNA Analysis to complete step 3B), case samples might be analyzed closer to the point of need, reducing the substantial delays associated with steps 1‐2) of the pipeline. There are, of course, substantial legal and regulatory hurdles that must be overcome for this vision to be achieved,^[^ ^32^ ^]^ which is beyond the remit of our team of science‐and‐engineering researchers. But we believed it would be of interest to explore whether a small‐footprint method could be developed to automate step 3A) up‐stream of Rapid DNA analysis, as a first step (of many) on a long road that may lead to this interesting outcome.

One option for automating step 3A) in forensic sample analysis is robotic workstations, which have been used to extract DNA from multi‐source forensic samples^[^ ^33^, ^34^ ^]^ including standard DE^[^ ^35^, ^36^ ^]^ as well as the modified DD procedure.^[^ ^3^ ^]^ But robotic workstations are large, and the robotic approach by nature handles batches of samples in parallel. These properties constitute a format that is well suited for laboratory use, but not for applications that require mobility and operation outside of the lab. An alternate approach to solving this problem is to use microfluidics.^[^ ^37^ ^]^ Specifically, microfabricated substrates containing arrays of enclosed microchannels have been used widely in forensic analysis,^[^ ^38^, ^39^ ^]^ and there is a growing body of literature^[^ ^38^, ^39^, ^40^, ^41^, ^42^, ^43^, ^44^ ^]^ describing the use of microfluidics to automate DE and DD and other parts of the sexual assault sample analysis pipeline. These techniques represent important advances; however, to date they have been reported at the “proof of concept” stage, applied to either i) testing simple mock samples (prepared in a lab), or ii) testing case‐like samples that are collected post‐coitus, but not demonstrating compatibility with full STR analysis and genotyping. The former is a particularly important point – post coital vaginal swabs containing sperm are challenging to process and analyze. It has been shown^[^ ^45^ ^]^ that techniques that can process simple mock samples do not necessarily work with real specimens collected post‐coitus, because of exposure of the latter to the harsh vaginal biochemical environment.

Here, we introduce a new solution to the problem of integrating, automating, and miniaturizing step 3A) in the processing of sexual assault samples, with a focus on STR analysis of vaginal swabs collected post‐coitus. The new method relies on digital microfluidics (DMF), a fluid handling technique in which samples are manipulated in a substrate without microchannels.^[^ ^46^, ^47^, ^48^ ^]^ Specifically, in DMF, droplets of fluid are manipulated by applying a series of electrical potentials to an array of electrodes that is covered with a dielectric layer and a hydrophobic layer. The electrostatic forces that are generated can be made to move, dispense, split, and mix droplets on the array, and the technique is called “digital” because the space over each electrode either has (1) or does not have (0) a droplet (a “bit”), and each bit is individually addressable. In the past, DMF has been used to automate processes including ELISA and sample processing for genome sequencing, both of which require many sequential reagent delivery and wash steps,^[^ ^49^, ^50^, ^51^, ^52^, ^53^ ^]^ generating “laboratory quality” results even in remote settings far from the laboratory (e.g., a refugee camp accessible only by charter flights operated by the United Nations^[^ ^54^ ^]^). The programmability inherent to DMF^[^ ^55^, ^56^ ^]^ led us to hypothesize that it could be well suited to automate DD, which includes a long list of steps that must be carried out with high precision^[^ ^30^ ^]^ in terms of volume, time, mixing efficiency, and temperature.

In the sections that follow, we first describe the development of a DMF‐assisted protocol for DD (DMF‐DD). Second, we describe the performance of this technique as applied to i) buccal swabs from female subjects spiked with semen, and ii) vaginal swabs collected post‐coitus. Finally, we describe the generation of STR profiles from samples processed in this manner using both conventional laboratory methods and the ANDE 6C Rapid DNA System. We propose that this advancement is a useful first step toward eventual implementation of sexual assault sample analysis outside of the lab, which could greatly reduce the time required to collect these important results.

## 2 Results

**Figure 1:**
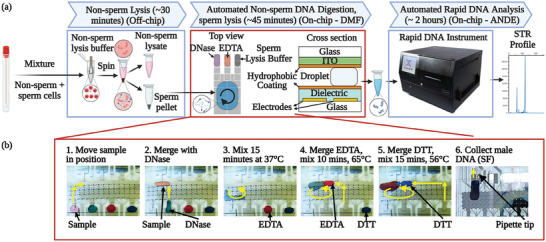
Differential digestion on DMF. a) Schematic (partly created with BioRender.com) showing the DMF‐assisted differential digestion protocol (DMF‐DD). First (in steps that are implemented off‐chip), non‐sperm cells are selectively lysed using non‐sperm lysis buffer (that does not contain DTT), and then the sperm cells are pelleted by centrifugation. Second (in steps that are implemented on‐chip), the resuspended sperm pellet is loaded into the DMF chip and processed. Third, the male fraction of DNA is purified, amplified and then characterized by STR analysis automatically using ANDE^TM^ 6C Rapid DNA System using the ANDE I‐Chip. b) Images (1) to (6) are frames from Video S1 (Supporting Information) demonstrating a portion of the steps that are automated by DMF (with dyes included for visualization). The following steps are performed automatically by activating electrodes in succession: 1) move sample (pink) from reservoir, 2) merge DNase (green) with the sample, 3) mix by moving in circles continuously for 15 min at 37 °C, 4) add EDTA (red) and mix for 10 min at 65 °C, 5) add DTT and proteinase K (blue) and mix for 15 min at 56 °C, and 6) collect DNA for analysis.

As a first step toward automating the sample processing of mixed‐source sexual assault samples in forensic applications, a digital microfluidic‐assisted method was developed for differential digestion of a sample stored on a swab (e.g., a vaginal swab collected from a sexual assault victim). The new DMF‐DD method is illustrated in **Figure** **1**a, and comprises key steps including non‐sperm lysis, non‐sperm DNA digestion, sperm lysis, DNA extraction and purification, and DNA analysis. A script was written in the open‐source MicroDrop control software^[^ ^48^ ^]^ to automate 8 steps in a 13‐step protocol (described in detail in the Methods section and summarized in Figure S1, Supporting Information) by serially activating sets of electrodes and activating controlled heaters and cooling fans.^[^ ^57^ ^]^ As shown in Figure 1b and Video S1 (Supporting Information), the DMF procedure, which has carefully controlled reagent volumes, incubation times, temperatures, and mixing conditions, can be completed in ≈45 min.

### 2.1 Automating Differential Digestion using DMF

DD is not used as widely by the forensic community as the “gold standard” of DE. Thus, as a “proof of concept” step (before introducing microfluidics), we evaluated the effectiveness of DD in comparison to DE in processing buccal swabs containing cheek cells from a female volunteer that were spiked with semen at dilution ratios selected to approximate the amount of sperm expected in a sexual assault sample. These samples were evaluated with PowerQuant System (Promega), a qPCR kit that reports concentration [A] of autosomal DNA (in a locus expected to be found in male and female cells), concentration [Y] of Y‐chromosome DNA (expected to be found only in male cells), and concentration [D] of autosomal DNA in a long locus that is particularly susceptible to degradation (expected to be found in male and female cells). These data, along with comparisons of the mass of male DNA spiked to male DNA recovered, were used to evaluate DE and DD according to metrics of purity, extraction efficiency, and degradation, as defined and explained in detail in Note S1 (Supporting Information) and summarized below.

The most important metric for this application is the purity of the male DNA found in the extract. Here, purity was defined as the ratio [A]/[Y], in which a high [A]/[Y] is indicative of a sample containing female DNA or a mixture of male and female DNA, while a low [A]/[Y] suggests a relatively pure male DNA sample. This method is imprecise, and values of 0.5–2 are typically accepted as evidence of purity.^[^ ^58^ ^]^ For the results collected here, in the non‐sperm fraction (NSF), both DE and DD had high [A]/[Y] ratios, which means that the NSF is predominantly female, as shown in Figure S2 (Supporting Information). More importantly, in the extracted sperm fraction (SF), the [A]/[Y] ratios for both techniques were below 2 (Figure S2, Supporting Information), suggesting that both methods result in relatively pure male DNA for analysis.

A second important metric for this application is extraction efficiency, defined as the amount of male DNA recovered divided by the amount that was spiked. As shown in Figure S2 (Supporting Information), DD was clearly superior in our experiments, with extraction efficiencies of ≈27%, relative to those of DE (≈8%–9%), a result that is comparable to Voorhees et al.^[^ ^24^ ^]^ who reported sperm extraction efficiencies between 5% and 23%. There are many potential explanations for this result. For example, the compositions of the non‐sperm lysis buffers in the DE and DD protocols used here are different, and some of the incubation steps in the DE method are longer than those of thecomparable steps in the DD technique. Perhaps these differences result in more sperm cells being lysed prematurely during the non‐sperm lysis step of DE in comparison to DD (noting the lower [A]/[Y] observed for the NSF in DE in Figure S2, Supporting Information). Most importantly, the extraction efficiency of DD was found to be greater than that of DE, which bodes well for downstream analysis.

A third key metric for this application is the potential degradation of sperm DNA by (unwanted) DNase activity. This is particularly important for DD, in which DNase is added to the sperm fraction (noting that in DE, non‐sperm DNA is removed by wash steps instead of DNase). Here, degradation was defined as the ratio [A]/[D], in which (as indicated by the manufacturer) [A]/[D] > 2 is expected for conditions in which DNA is degraded.^[^ ^58^ ^]^ The results of these experiments (Figure S2, Supporting Information) suggest modest evidence of DNA degradation in the NSF (which is not important for the current application). But much more importantly, the data in Figure S2 (Supporting Information) suggests that there is no appreciable degradation in the SF for either DD or DE.

We then scaled the DD method down to implement it by digital microfluidics, as illustrated in Figure 1. Figure S3 (Supporting Information) shows the results of head‐to‐head tests comparing manual DD to DMF‐DD. The results confirm that automating DD using DMF is feasible, and that the DMF‐DD method generates results comparable to manual processing, with high purity, high recovery, and low degradation for the sperm fraction. In sum, proof‐of‐concept tests with spiked/mixed buccal swab samples suggest that DNA generated by the DMF‐DD procedure has desirable characteristics for downstream analysis.

### 2.2 Application to Human Post‐Coital Samples

The new method was then applied to samples that are close analogues to forensic samples tested in sexual assault cases.^[^ ^45^ ^]^ Specifically, eleven vaginal swabs were collected at different time‐points post‐coitus (PC; 1, 3, 6, 12, 24, 48, 72 h). As a first test, the samples were evaluated with the PowerQuant System, with results shown in Table S1 (Supporting Information). As expected, [A]/[D] < 2 for all sperm fractions tested, indicating that the DNA in the SFs were not degraded. Furthermore, as highlighted in Figure S4 (Supporting Information), high (male DNA) purity was observed for the extracts isolated from samples collected between 1–48 h PC.

**Figure 2:**
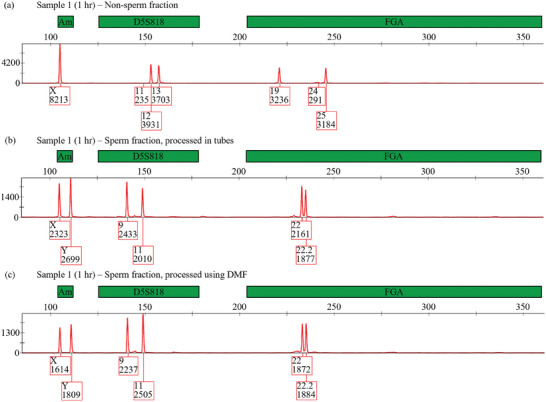
Representative electrophoretic analysis of sample 1, a vaginal swab collected 1 h post‐coitus, generated using standard laboratory analysis techniques. a) Partial electropherogram of fluorescence intensity (red trace) as a function of fragment length (bp) of the non‐sperm fraction. Labels above the plot (green filled boxes) indicate which markers the detected peaks correspond to, including the sex‐typing marker Amelogenin (“Am”) and STR loci D5S818 and FGA. Labels below the plot (red outlined boxes) indicate the number of repeats for STR loci or X/Y designation for sex typing, as determined using GeneMapper^TM^ ID‐X genotyping software (Applied Biosystems), as well as the peak height in rfu. b) Partial electropherogram of sperm fraction processed by manual DD. c) Partial electropherogram of sperm fraction processed using DMF‐DD.

The samples were then evaluated by STR analysis. In initial tests, Samples 1–3 (1, 3, and 6 h PC, respectively) were evaluated using standard laboratory techniques, with discrete steps for extraction, cleanup, amplification and electrophoretic analysis. In Sample 1, as an extra test for manual versus DMF processing, after removing the non‐sperm lysate, the sperm pellet was resuspended and then split into two equal volumes, which were processed by manual DD (labeled “SF*”) or DMF‐DD (labeled “SF”). **Figure** **2** shows representative partial electropherograms for Sample 1, showing one of the fluorescent channels for the non‐sperm fraction (NSF), the sperm fraction processed manually (SF*), and the sperm fraction processed by DMF‐DD (SF). In these data (and in other STR electropherograms), peak height is a rough indicator of analyte concentration, with subtle differences attributed to variations in the kinetics of amplification and band broadening. The higher peak heights in the NSF data relative to the SF data indicates the prevalence of female DNA in the sample, illustrating the need for extraction. Most importantly, the peak assignments line up with expectations for this sample, with the NSF exhibiting a female marker for sex‐typing, 11 and 13 repeats for the D5S818 locus, and 19 and 25 repeats for the FGA locus, and the SF exhibiting male markers for sex‐typing, 9 and 11 repeats for the D5S818 locus, and 22 and 22.2 repeats for the FGA locus.

Complete electropherograms (including all fluorescent channels) for all fractions of samples 1 to 3 are shown in Figures S5–S11 (Supporting Information). As indicated, equivalent STR profiles were achieved whether sample was processed manually (for sample 1) or using DMF (for all three samples). While there are previous reports^[^ ^38^, ^39^, ^40^, ^41^, ^42^, ^43^, ^44^ ^]^ of microfluidic methods applied to sexual assault sample processing and analysis for spiked samples, the data in Figure 2 (and Figures S5–S11, Supporting Information) uniquely represent STR results generated from post‐coital vaginal swabs using a microfluidic technique.

### 2.3 Application to Human Post‐Coital Samples with Rapid DNA Analysis

**Figure 3:**
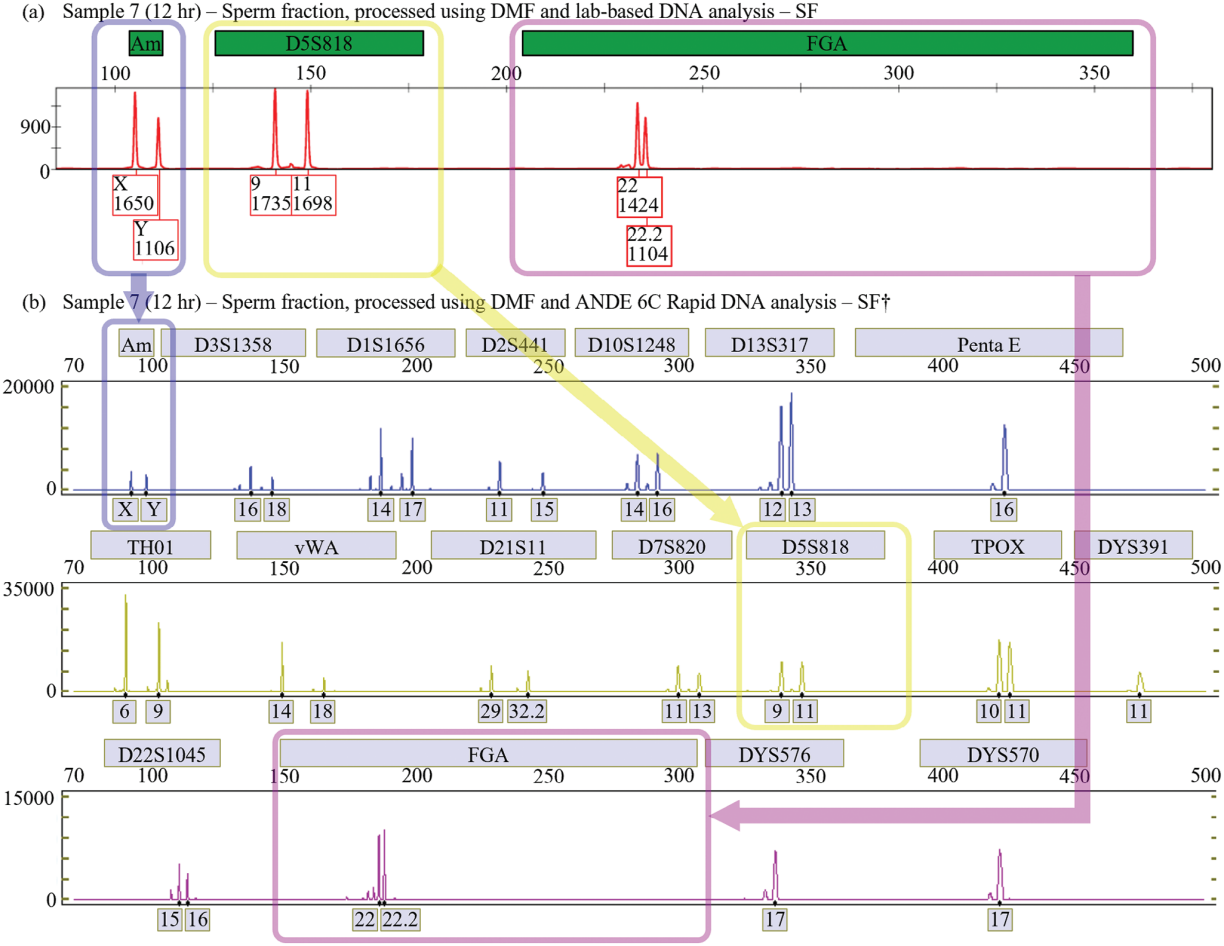
Representative electrophoretic analysis of sample 7, a vaginal swab collected 12 h post‐coitus, by standard laboratory analysis techniques and Rapid DNA analysis. a) Partial electropherogram of fluorescence intensity (red trace) as a function of fragment length (bp) of the DMF‐DD sperm fraction, generated using standard laboratory analysis techniques. Labels above the plot (green filled boxes) indicate which markers the detected peaks correspond to, including the sex‐typing marker Amelogenin (“Am”), and the STR loci D5S818 and FGA. Labels below the plot (red outlined boxes) indicate either the number of repeats for STR loci or X/Y designation for sex typing as determined using GeneMapper^TM^ ID‐X genotyping software (Applied Biosystems), as well as the peak height. b) Partial electropherograms of fluorescence intensity (blue, yellow, and purple traces) as a function of fragment length of the DMF‐DD sperm fraction generated using the ANDE^TM^ 6C system. Labels above the three plots (gray filled boxes) indicate the markers the detected peaks correspond to, and labels below the plot (gray filled boxes) indicate either the number of repeats for STR loci or X/Y designation for sex typing. Blue, yellow, and purple boxes (and arrows) indicate loci in a) that correspond to loci in b): Am, D5S818, and FGA, respectively.

To evaluate suitability of DMF‐DD for processing samples upstream of Rapid DNA analysis, in samples 4–11 (1, 3, 6, 12, 24, 24, 48, and 72 h PC, respectively), after collecting the sperm lysate from the DMF chip, the processed sample was split into two equal volumes, SF and SF†. The SF fractions were evaluated as described in the previous section using conventional lab equipment and personnel, while the SF† fractions were processed using an ANDE 6C instrument, which allowed for automated purification, amplification and electrophoretic analysis in under 2 h. **Figure** **3** shows representative partial electropherograms for the SF (conventional laboratory analysis), and the SF† (Rapid DNA analysis) from sample 7. Peaks for amelogenin, D5S818, and FGA are highlighted in this data, illustrating how the two types of analyses (standard laboratory versus Rapid DNA) identify the same alleles.

Complete electropherograms (for both kinds of analysis) for all fractions of Samples 4–11 are shown in Figures S12–S35 (Supporting Information). As indicated, the STR profiles generated by conventional lab techniques and Rapid DNA analysis were quite similar, noting that the conventional laboratory analysis used here tests for 16 loci, while the ANDE system tests for 27. In sum, the DMF‐DD sample processing method is compatible with Rapid DNA STR analysis, suggesting the potential for integrated methods relying on both techniques in the future.

### 2.4 Influence of Post‐Coital Interval on Performance of DMF‐DD

As described above, DE/DD is applied to sexual assault case samples from mixed sources in an attempt to generate “single source” STR results that can be matched to a reference sample. Thus, the STR analyses from the eleven post‐coital vaginal swab samples processed by DMF‐DD (Figures 2 and 3; Figures S5–S35, Supporting Information) were compared with data generated from buccal swabs from the male and female partners in the couple. **Table** **1** summarizes the results. As shown, all 16 loci from the male subject tested by conventional laboratory methods and at least 25 of the 27 loci tested by the ANDE system were correctly identified for samples collected up to and including 48 h after coitus. Interestingly, this is the same range of samples that qPCR analysis indicated had high male DNA purity (Figure S4, Supporting Information).

**Table 1:** Comparison of STR results for sperm fractions extracted using DMF‐DD from post‐coital samples relative to reference samples. Sperm fractions (SF) (no dagger) were processed using DMF and standard laboratory analysis, sperm fractions (SF†) (dagger) were processed using DMF and rapid DNA analysis. The number of loci identified from the male partner and female partner are indicated for each sample.

Sample	PC Interval	SF (lab‐based)			SF† (Rapid DNA)		
		Type	#male loci	#female loci	Type	#male loci	#female loci
1	1 h	Single	16	0			
2	3 h	Single	16	0			
3	6 h	Single	16	0			
4	1 h	Single	16	0	Single	27	0
5	3 h	Single	16	0	Single	27	0
6	6 h	Single	16	0	Single	27	0
7	12 h	^‡^	16	3	Single	27	0
8	24 h	Mixture	16	16	Mixture	26	23
9	24 h	Mixture	16	15	Mixture	27	3
10	48 h	Mixture	16	6	Single	25	0
11	72 h	Mixture	16	16	Mixture	8	24

^‡^ Although technically a “mixture” because of three sets of peaks corresponding to female alleles (D3S1358, D13S317, and D19S433), the peak heights are so small that an analyst might alternately label the sample to be “single,” which is indeed what the ANDE Expert System Software labels it.

A deeper dive into the comparison between the processed post‐coital samples and male reference sample suggests that the data support a clean “single source” assignment to the male subject for sperm fractions extracted from samples collected up to and including 12 h PC (i.e., Samples 1–7), while samples collected 24, 48, or 72 h after coitus were identified as a “mixed source” (i.e., Samples 8–11). This is not surprising, given the well‐known relationship between the amount of time that passes after coitus (or sexual assault) and reduced numbers of sperm^[^ ^59^ ^]^ and/or degraded seminal fluid^[^ ^45^ ^]^ and DNA,^[^ ^30^ ^]^ which can make identification less robust. While “single source” data is preferred, “mixture” profiles can be interpreted using deconvolution techniques.^[^ ^60^ ^]^

**Figure 4:**
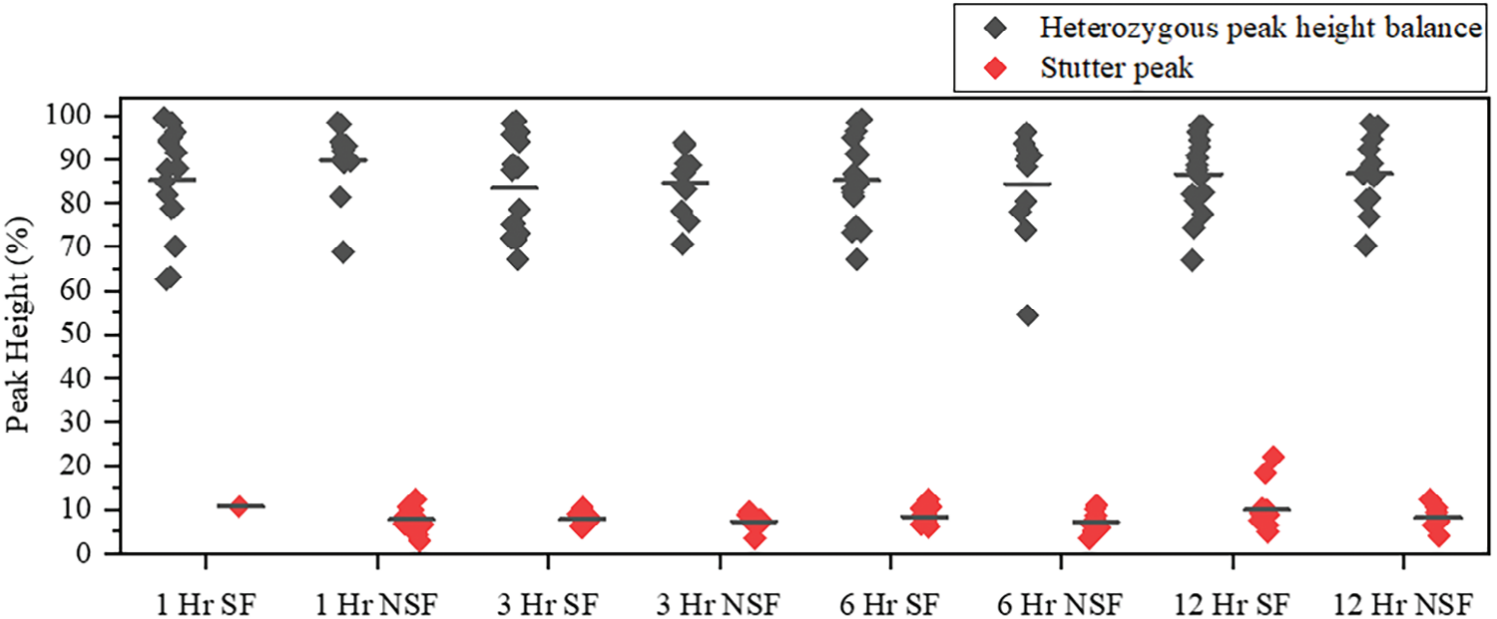
STR Quality Analysis. PCR product characterization for samples 4 to 7 (collected 1, 3, 6, and 12 h post‐coitus), using the electropherograms generated by conventional laboratory analysis. Heterozygous peak height balance (black diamonds) was calculated by dividing the smaller peak height of the two alleles by the larger one. Stutter peak (red diamonds) was calculated by dividing the stutter height by the larger of the peak heights for the corresponding allele at the same locus.

To verify that the sperm fractions extracted using DMF‐DD from samples collected up to 12 h PC were really single source, we evaluated heterozygous peak height balance and stutter peak height^[^ ^61^ ^]^ for each of these samples. Briefly, heterozygous peak height balance of less than 70% can be indicative that a sample may contain DNA from multiple sources; as shown in **Figure** **4**, the average peak height balance was 80% or higher for all samples collected up to 12 h PC. Stutter peak height is a measure of a common artifact in STR analysis whereby the PCR reaction does not proceed perfectly, resulting in deletion or insertion of one (or more) repeat units – these products are detected at a different time point than the expected allelic DNA.^[^ ^62^ ^]^ Stutter peak heights of less than 15% are typical of high quality STR analysis of single‐source DNA, while stutter heights larger than 15% can be indicative of mixtures or poor quality DNA.^[^ ^61^ ^]^ As shown in Figure 4, the average stutter peak heights were below this threshold for samples collected up to 12 h PC. Thus, even for the samples tested here collected after some delay (i.e., 6 and 12 h PC), these profiles are consistent with single‐source profiles.

The trends in Figure 4 bode well for potential future applications of the DMF‐DD method combined with “Rapid DNA” analysis outside of the laboratory for samples collected within 12 h post‐coitus. Additional work is needed to accommodate samples collected at longer durations post coitus; additional samples and experiments are needed to verify the trend of analysis as a function of time PC, as well.

## 3 Discussion

The bulk of the data presented here was generated via microfluidic processing of post‐coital samples – a sample type that is very close to case samples from victims of sexual assault – combined with full STR analysis. As described above, while there are several previous reports of automated processing of sexual assault samples using microfluidics,^[^ ^38^, ^39^, ^40^, ^41^, ^42^, ^43^, ^44^ ^]^ these reports either used samples that are not representative of case samples (e.g., buccal swabs from female subjects spiked with semen) or did not show full STR analysis. Spiked buccal swabs are much more readily accessible than post‐coital swabs and can be a useful tool for demonstrating proof‐of‐concept for new techniques. And in fact, we also used buccal swabs spiked with semen to demonstrate some of the proof‐of‐concept results reported herein. But spiked buccal swabs are simply not a close enough proxy for sexual assault case samples, which motivated us to challenge the new DMF‐DD method with vaginal swabs collected post‐coitus.

Post‐coital vaginal swab samples are impacted by biochemical processes that are particularly harsh toward sperm cells. First, neutrophils in the vaginal tract may phagocytose sperm (either fully or partially).^[^ ^45^ ^]^ Buccal swabs do not contain live neutrophils, so this effect is not recapitulated in buccal swabs spiked with semen. Second, the vaginal environment degrades proteins and other molecules present in the seminal fluid that normally protect sperm cells; with degradation, this protective effect is stymied, such that female DNA can adhere strongly to the sperm cells (making extraction more challenging).^[^ ^45^ ^]^ Likewise, the binding of external DNA to sperm induces apoptosis, which degrades the male DNA within.^[^ ^45^ ^]^ Third, absent other effects, storage in a wet/warm/mucosal environment is known to enhance degradation of male DNA relative to storage of a dry buccal swab.^[^ ^63^ ^]^ Fourth, even if the sperm is not completely lysed, exposure to the vaginal environment can cause the cellular membrane to become weakened, such that the sperm is susceptible to premature lysis during the non‐sperm lysis step of DE/DD.^[^ ^31^ ^]^ In sum, the types of complexities that are expected in sexual assault case samples are only fully recapitulated using vaginal samples collected post‐coitus.^[^ ^45^ ^]^ With this in mind, the new DMF‐DD method described here was challenged by application to processing eleven separate vaginal samples collected post‐coitus after a range of different intervals.

As noted in the introduction, forensic sexual assault case samples are typically 1) transported, 2) queued, 3A) processed to separate multiple sources (if needed), and 3B) analyzed. Here we focused on automating and miniaturizing step 3A), with a special interest in determining whether the technique was compatible with analysis in step 3B) by a Rapid DNA analyzer. The data presented here demonstrate feasibility, suggesting the potential to someday carry out steps 3A) and 3B) in the field, potentially bypassing the bottleneck of steps 1‐2). While our primary goal was to confirm analytical performance for 3A), we have also begun experimenting with reducing the run‐time for this step. As described in Note S2 (Supporting Information), we carried out a chemometric optimization scheme that involved processing more than 50 buccal swabs spiked with semen (Tables S2 and S3, Supporting Information), which yielded an optimized DMF‐DD method (Figure S36, Supporting Information) that reduced the DMF run time from 45 to 5 min, without sacrificing performance. This result must be validated in the future by application to vaginal swab samples collected post‐coitus.

Finally, we acknowledge that the methods reported here remain a work‐in‐progress, as they still require manual steps that are not ideal for point‐of‐care applications. In the future, we propose to build from examples in the literature to develop modules for automated swab extraction,^[^ ^64^ ^]^ microfluidic sperm isolation,^[^ ^65^, ^66^ ^]^ and a direct interface to the Rapid DNA analysis, either in a combined system or via a DMF‐Rapid DNA interface (similar to interfaces described previously for other instruments^[^ ^67^ ^]^). Additionally, we propose to develop cartridges with pre‐stored reagents (following approaches described for other DMF applications described previously^[^ ^68^ ^]^), such that the user can simply load the sample, press a button, and be done. These advances would form the basis of a sample‐to‐answer system that could be operated outside of the laboratory, representing additional steps toward a fieldable method that could be operated in a medical clinic or police station (settings where the ANDE instrument is in use today).

## 4 Conclusion

A differential digestion procedure for forensic sexual assault case samples was automated using digital microfluidics, reducing a complex process from 13 to 5 manual steps. The method was first applied to evaluating buccal swabs spiked with semen, allowing assessment of extraction efficiency, sperm fraction purity and DNA degradation. The method was then applied to processing vaginal swabs collected post‐coitus, followed by STR analysis using both standard laboratory procedures and a Rapid DNA System. Non‐sperm fractions had minimal male DNA present, confirming that sperm cells did not prematurely lyse to any great extent. Sperm fractions extracted from post‐coital swabs, collected up to 12 h after coitus resulted in well balanced, single source male STR profiles for both manual and DMF protocols. Sperm fractions extracted from post‐coital swabs collected 24 to 72 h post‐coitus resulted in mixture profiles that (where permitted) can be interpreted using deconvolution techniques. In sum, the DMF‐DD method can produce STR profiles that can be used to identify the sperm donor in post‐coital vaginal swabs. By showing compatibility with the ANDE 6C System this opens the door to future methods allowing for sample‐to‐answer automated testing of sexual assault samples.

## 5 Experimental Section

Unless otherwise stated, reagents were acquired from Sigma (Oakville, ON), and all aqueous solutions were formed in nuclease‐free ultrapure distilled water. Chrome on glass substrates coated with AZ1500 photoresist were from Nanofilm (Westlake Village, CA) (5‐inch by 5‐inch) and Telic Company (Valencia, CA) (3‐inch by 3‐inch). ITO‐coated glass slides (25 × 75 mm, 8 ohm sq.^−1^) were purchased from Riley Supplies. AZ400K, CEP, and MF312 were from Kayaku Advanced Materials. FluoroPel PFC 1104 V and PFC 110 solvent were purchased from Cytonics, LLC (Beltsville, MD). Sterile cotton swabs (258062WC) used for buccal samples were purchased from Hardy Diagnostics. Sterile cotton swabs (FS22363584) used for vaginal samples were purchased from Fisher Scientific. Semen from a Caucasian 36‐year‐old male was purchased from Lee Biosolutions and was stored at –20 °C in 100 µL aliquots. Aqueous dithiothreitol (DTT) solution (1.0 M pH 8.0) was purchased from Independent Forensics (Lombard, IL). Aqueous Tris‐NaCl‐EDTA (TNE) buffer was purchased from Quality Biological. DNase 1 kits were acquired from Fisher Scientific (Burlington, ON), containing aqueous DNase 1 solution (1 U µL^−1^) in a storage buffer (50 mm Tris‐HCl (pH 7.5), 10 mm CaCl_2_ and 50% glycerol) and 10X reaction buffer (100 mm Tris‐HCl pH 7.5, 25 mm MgCl_2_, 1 mm CaCl_2_). QIAamp DNA Investigator kits were purchased from Qiagen (Germany), including Proteinase K solution (20 mg mL^−1^), AL buffer, ATE buffer, and a carrier RNA solution that is prepared to 1 µg µL^−1^ as per manufacturer's instructions. PowerQuant System and amplification‐grade distilled water were purchased from Promega Corporation (Madison, WI).

### Digital Microfluidic Chip Fabrication

The bottom‐plate photomask was designed using AutoCAD, then fabricated in‐house at the Centre for Research and Applications in Fluidic Technologies (CRAFT). A 5‐inch by 5‐inch chrome on glass substrate coated with AZ1500 photoresist was exposed using a uPG pattern generator (Heidelberg) followed by develop (AZ400:water 1:4, 1 min), etch (CEP, 1 min), and strip (AZ400K, 5 min) steps with a deionized (DI) water rinse between steps, and drying by N_2_ at the end.

DMF bottom plates were fabricated on 3‐inch by 3‐inch chrome on glass substrates coated with AZ1500 as described previously.^[^ ^48^, ^54^ ^]^ Briefly, substrates were exposed through the mask using a mask aligner (OAI, Milipitas, CA) for 3 s at 22.8 mW cm^−2^ (measured using OAI's Model 308 power meter at 365 nm), followed by develop (MF312:water 1:1, 25 s), etch (CEP, 1 min 45 s), and strip (AZ400K, 5 min) steps, with a DI water wash between steps and drying by N_2_ at the end. When complete, the pattern featured 104 roughly square (2.2 × 2.2 mm) driving electrodes (where 84 of them formed a 4 row × 21 column electrode array) and 10 loading electrodes or ‘reservoirs’ (6.16 × 5.81 mm), which each connected to an electrode pad. The electrode pads were then covered with dicing tape, and ≈6 µm parylene‐C was deposited using the CS 2010 Parylene Coater in the Toronto Nanofabrication Centre (TNFC).

Fluoropel solution (1%) was prepared by diluting FluoroPel PFC 1104 V in PFC 110. This solution was deposited on the Parylene‐C‐coated bottom plate substrates by spin coating at 1500 rpm for 30 s followed by a 120 °C bake in an oven for 10 min. This solution was also deposited on ITO‐glass top‐plate substrates by dip coating followed by a 160 °C bake in an oven for 10 min. Finally, each digital microfluidic chip was assembled by joining a bottom and top plate with a ≈360 µm‐thick spacer formed from 4 layers of double‐sided tape (3M) [thickness measured using a 150 mm digital caliper (Sparkfun Electronics, Niwot, CO)].

### Control Hardware

Digital microfluidic chips were controlled by interfacing to a Zed‐box,^[^ ^57^ ^]^ an updated version of previous versions of the open‐source Dropbot.^[^ ^48^, ^54^ ^]^ A critical feature of the Zed‐box for this application is the integrated PID‐driven temperature control system.^[^ ^57^ ^]^ Chips were interfaced to the Zed‐box through a pogopin connector, and electrodes were actuated in preprogrammed steps which allowed droplet dispensing, moving, and mixing by applying (typically) 100 V_RMS_ as square waves at 10 kHz, conditions observed to generate driving forces below the saturation force for the fluids manipulated here.^[^ ^69^ ^]^

The heaters were resistive elements housed underneath the chip, and thermistors were used to provide feedback. There was also a built‐in fan to expedite cooling when the actual temperature is higher than the set temperature.

### Sample Collection and Preparation

Buccal swabs were collected and processed with informed consent according to Protocol # 00 036059 approved by the University of Toronto Research Ethics Board. Briefly, a female volunteer sampled the inside of both cheeks for 5 to 10 s using sterile cotton swabs, which were then dried overnight in loosely capped 15 mL centrifuge tubes. A commercial semen sample was thawed at room temperature then diluted with PBS at ratios of 1:10 and 1:100. 50 µL of diluted sperm suspension was added to the dried buccal swabs and placed back into the same 15 mL centrifuge tube and left to dry overnight prior to extraction and analysis (described below). Sperm densities in the diluted suspensions were determined by hemacytometer and phase contrast microscopy (Nikon Ni‐E Eclipse, 20X magnification), allowing estimation of the numbers of sperm cells added to the swabs to be 3 × 10^5^ and 3 × 10^4^ for the 1:10 and 1:100 dilutions, respectively.

Post‐coital vaginal swabs were collected and processed with informed consent according to the same protocol (# 00 036059). Briefly, vaginal swabs were collected 1, 3, 6, 12, 24, 48, or 72 h post‐coitus (from the same pair of donors), and were dried overnight in loosely capped 15 mL centrifuge tubes. For both types of samples, swabs were stored in loosely capped tubes at room temperature for short periods (days) or at −20 °C for long periods.

### Differential Extraction

Differential extraction was performed following the method described by Alderson et al.^[^ ^28^ ^]^ with some modifications. Briefly:

*Aqueous cell collection*: The cotton‐tip of a swab was cut into a ClickFit tube (Promega) containing 400 µL PBS, which was then vortexed, incubated at room temperature for 5 min, then centrifuged at 13 000 rpm for 3 min using DNA IQ spin basket (V1225, Promega). The aqueous extract was discarded, leaving behind ≈30 µL. The pellet (including sperm and non‐sperm cells) was resuspended in the fluid left behind.

*Non‐sperm lysis*: 505 µL of non‐sperm extraction buffer (395 µL TNE buffer, 50 µL 10% SDS, 50 µL water, 10 µL Proteinase K solution) was added to the ClickFit tube containing the resuspended pellet, and the swab was incubated at 56 °C for 1 h in a shaking drybath at 600 rpm (Thermofisher Scientific, Whitby, ON). The swab was then centrifuged at 13 000 rpm for 5 min using the spin basket. The pellet and supernatant were collected separately, and the spin basket with swab was discarded. That is, 300 µL of the supernatant was extracted and placed in a fresh tube containing 300 µL warm (37 °C) AL buffer. The remainder of the supernatant was discarded, leaving behind ≈30 µL into which the pellet of sperm cells was resuspended.

*Non‐sperm DNA removal from resuspended sperm*: Free floating DNA was removed from the sperm pellet using 3 wash steps: 1) 500 µL TNE added, the sample was centrifuged at 13 000 rpm for 5 min, and the supernatant was discarded, 2) 500 µL DI water added, the sample was centrifuged at 13 000 rpm for 5 min, and the supernatant was discarded, and 3) step 2) was repeated. ≈30 µL of supernatant was left behind with the pellet at the end of each wash step.

*Sperm lysis*: 400 µl sperm extraction buffer (150 µL TNE, 173.4 µL water, 40 µL 10% SDS, 15.6 µL 1 m DTT, 20 µL Proteinase K) was added to the ClickFit tube containing the washed sperm pellet, and the tube was incubated at 70 °C 900 rpm for 10 min. The sperm lysate was then transferred to a fresh tube containing 300 µL warm (37 °C) AL buffer and 1 µL carrier RNA.

*DNA purification*: The non‐sperm lysate and sperm lysate from above were further processed using the QIAamp DNA investigator kit (Qiagen) per manufacturer's instructions (which includes the use of a carrier RNA solution). Purified DNA was eluted in 50 µL ATE buffer and stored at −20 °C until analysis – these solutions were designated the Non‐Sperm Fraction (NSF) and sperm fraction (SF), respectively.

### Manual Differential Digestion

Manual differential digestion was performed using methods from Wong and Mihalovich^[^ ^3^ ^]^ with some modifications. The 13 steps are summarized in Figure S1(a) (Supporting Information). Briefly:

Aqueous cell collection was performed as described in the Differential Extraction sub‐section above.

**Steps 1–4: Non‐sperm lysis**. Swabs were incubated in 510 µL Tween 80 buffer (2% Tween 80, 20 mm Tris‐HCl, 1 mm EDTA) and 10 µL Proteinase K solution at 56 °C 600 rpm for half an hour, then centrifuged using a spin basket. Non‐sperm lysate was processed and purified (forming the NSF) as described in the Differential Extraction sub‐section above. The supernatant was removed, leaving behind ≈30 µL liquid into which the sperm pellet was resuspended. As a quality control step, the spin basket was visually inspected at the end of this process. In one sample, an unexpected sticky residue was visible in the spin basket, and this sample was excluded from further analysis.

**Steps 5–10: Non‐sperm DNA digestion of free DNA**. 150 µL Digestion buffer (20 µL DNase 1, 20 µL 10X reaction buffer and 160 µL DI water) was added to the suspended sperm cells and mixed gently by inverting the tube, followed by incubation at 37 °C for 15 min. 20 µL 0.5 m EDTA was then added followed by incubation at 65 °C for 10 min.

**Steps 11–13: Sperm lysis and purification**. 20 µL 1 m DTT and 10 µL Proteinase K were added to the processed sperm suspension followed by incubation at 56 °C, 900 rpm, for 15 min. This digest was transferred to a tube containing AL buffer and carrier RNA, and the DNA was purified as described in the Differential Extraction sub‐section above, forming the SF.

### Digital Microfluidic‐Assisted Differential Digestion

As summarized in Figure S1(b) (Supporting Information), the DMF‐assisted differential digestion method constitutes 13 steps, 5 of which were performed manually by the operator (labeled Steps 1 to 5), and the remaining 8 steps were performed automatically using DMF (labeled steps i to viii).

**Steps 1 to 4: Non‐sperm lysis (off chip)**. Aqueous cell collection and non‐sperm lysis were performed as described in the manual differential digestion sub‐section above to generate the NSF and a ≈30 µL suspension of the sperm pellet.

For some experiments, half of this suspension was transferred to a new tube (to be processed manually according to the manual differential digestion sub‐section above) and the other half was processed on the DMF chip as described below. For other experiments, the entire resuspended sperm pellet was processed using DMF.

**Step 5: Non‐sperm DNA digestion and sperm lysis (on‐chip)**. In typical experiments, 2 × 15 µL aliquots of resuspended sperm sample were loaded into adjacent reservoirs on a digital microfluidic chip. 15 µL digestion buffer (1:1:1 DNase 1: 10X reaction buffer: 0.2% Tween 80) was added to a third reservoir. A 4 µL aliquot of 0.25 m EDTA with 0.1% Tween 80 was loaded into a reservoir. 30 µL of sperm extraction buffer (1:2:3 Proteinase K, 1 m DTT, 0.2% Tween 80) was loaded into a reservoir.

The system was engaged, and the sperm sample aliquots were combined with the digestion buffer on the chip (step i), followed by step ii; a 15 min mixing routine at 37 °C. EDTA was then merged with the sample droplet (step iii), which was then mixed (step iv) and incubated at 65 °C for 10 min (step v). Sperm extraction buffer was then merged with the sample (step vi), mixed (step vii) and incubated at 56 °C for 15 min (step viii). Finally, the sperm lysate was moved to the collection reservoir and collected by pipette into a tube.

In experiments that involved buccal swabs spiked with semen and for samples 1 to 3, the sperm lysate collected from the chip was transferred to a tube containing AL buffer and carrier RNA, and the DNA was then purified to form the SF, as described in the Differential Extraction sub‐section above.

For samples 4 to 11, the sperm lysate was transferred to a tube, 70 µL ATE buffer was added and mixed by pipetting up and down 10 times. (Note that this unique step is not a common one, and was included simply to ensure adequate mixing for samples that were subsequently split for analysis by different methods. This is not required for eventual deployment in the field, in which case the entire sample would be processed using rapid DNA analysis.) Half the volume was then transferred to a tube containing AL buffer and carrier RNA and the DNA was purified, as described in the Differential Extraction sub‐section above to form the SF, for analysis by conventional laboratory methods. The other half of the volume was designated SF† and was deposited onto an ANDE Smart Swab and left to dry. The swab was then loaded into an I‐Chip and the ANDE 6C instrument for analysis.

### DNA Quantitation

PowerQuant (Promega) and Applied Biosystems QuantStudio 6 (Thermo‐Fisher) were used as per manufacturer's instructions and as described previously.^[^ ^58^ ^]^ Briefly, the male DNA standard from the kit (50 ng µL^−1^) was diluted serially in dilution buffer to create DNA standards containing 2, 0.08, and 0.0032 ng µL^−1^ of male DNA. 2 µL of standards were added to 18 µL of reaction mixture (7:10:1 amplification grade water: PowerQuant 2X Master Mix: PowerQuant 20X Primer/Probe/IPC Mix) in an Applied Biosystems MicroAmp Fast Optical 96 Well Reaction Plate, 0.1 mL (ThermoFisher) – these reactions were used to generate the standard curve. In lieu of DNA, the negative control had 2 µL of amplification grade water. 2 µL of samples with unknown amounts of DNA extracted from samples manually and by DMF as described above were added to 18 µL of reaction mixture (in the same 96‐well plate). Duplicate wells were prepared for each sample. The plate was centrifuged at 2500 rpm for 1 min to remove all bubbles then loaded into the QuantStudio 6. The amplification protocol had a hold stage at 98 °C for 2 min, then 39 cycles of 98 °C for 15 s, 62 °C for 35 s, with a ramp rate between all steps of 2.44 °C s^−1^, for a total of 1 h.

After running the PCR, data was exported and analyzed using Promega PowerQuant Analysis Software. The amount of DNA quantified was reported as a concentration, [A], [Y], or [D] as described in Note S1 (Supporting Information). Absolute amounts of DNA were obtained by multiplying the concentration by the eluate volume, 50 µL.

### Lab‐based DNA Analysis

NSF and SF samples generated as described above were evaluated as described previously.^[^ ^28^ ^]^ Briefly, STR amplification was performed using the AmpFLSTR Identifiler Plus DNA amplification kit (Thermo‐Fisher, Foster City, CA) using the GeneAmp PCR System 9700 (Thermo‐Fisher). Capillary electrophoresis was performed using the 3500XL Genetic Analyzer (Thermo‐Fisher), with 1.2 kV, 12 s injection. Allele calls for Table 1 were assigned as follows. First, all peaks identified by Genemapper ID‐X were recorded. Peak heights were then assessed, and minor peaks at stutter positions with intensity less than 15% of the corresponding major peak were considered stutter and were removed from the list. Minor peaks that were not at stutter positions remained on the list, regardless of their peak heights. Finally, the number of alleles that corresponded to known alleles from the male and female subjects were (separately) summed to arrive at # male loci and # female loci, respectively.

### Rapid DNA Analysis

Samples were analyzed using the ANDE I‐Chip in the ANDE 6C instrument as described previously.^[^ ^9^ ^]^ Briefly, in this system, DNA is extracted using chaotic bubbling, purified using a silica membrane, and then exposed to STR amplification reagents (the ANDE FlexPlex27 kit) followed by electrophoretic separation and detection. ANDE Expert System software version 2.0.6^[^ ^9^ ^]^ was used for allele calls. Allele calls for Table 1 were assigned as those indicated by the ANDE Expert System (“as is” without considering peak intensities). The number of alleles that corresponded to known alleles from the male and female subjects were (separately) summed to arrive at # male loci and # female loci, respectively.

### STR Quality Analysis

Electropherograms produced by the 3500XL Genetic Analyzer were analyzed following approaches described previously.^[^ ^61^ ^]^ For each section of the electropherogram representing a particular locus, the largest two peaks (allelic peaks P1 and P2, where P1 is the peak with the highest intensity) were used to calculate the peak balance, and any other peaks (P3, P4, etc.) were used to calculate stutter height. Peak balance was calculated as P2/P1. Stutter height for P3 was calculated as P3/P1.

## Conflict of Interest

M.S. and J.L.F. are employees of ANDE Corporation (manufacturer of the ANDE 6C Rapid DNA System used herein, and co‐funder of the study).

## Author Contributions

M.E., J.M., J.L.F., and A.R.W. conceived the concept of developing a DMF‐driven protocol that can be performed outside of the lab and is compatible with a Rapid DNA System. M.E. and L.B. fabricated DMF chips, performed manual differential extraction and differential digestion, DMF differential digestion, and performed real time PCR to quantify the DNA. C.G., P.K., A.D., and V.L. developed and implemented the chemometric optimization of DMF‐DD. A.G., A.G.C., and B.E. performed lab‐based STR analysis, and the data was reviewed by B.E. and J.M. M.S. operated the Rapid DNA System. M.E. and A.R.W. wrote and edited the manuscript. All authors discussed the results and commented on the manuscript.

## Supporting information

Supporting Information

Supplemental Video 1

## Data Availability

The data that support the findings of this study are available in the supplementary material of this article.
