# Supplementary material for: Toward Analysis at the Point of Need: A Digital Microfluidic Approach to Processing Multi‐Source Sexual Assault Samples
Source: Adv Sci (Weinh). 2024 Sep 4;11(41):2405712. doi: 10.1002/advs.202405712 (PMC11538644; doi:10.1002/advs.202405712)
Supplement: Supplementary file 1 — Supporting Information [file ADVS-11-2405712-s001.docx]

Supporting Information for

**Towards Analysis at the Point of Need: A Digital Microfluidic Approach to Processing Multi-Source Sexual Assault Samples**

*Mohamed Elsayed, Leticia Bodo, Christine Gaoiran, Palig Keuhnelian, Advikaa Dosajh, Vivienne Luk, Melissa Schwandt, Julie L. French, Alpana Ghosh, Barbara Erickson, Amanda G. Charlesworth, Jonathan Millman, and Aaron R. Wheeler**

**Table of Contents**

[Supporting Note S1 1](#_Toc164460121)

[Supporting Note S2 3](#_Toc164460122)

[Supporting Video 5](#_Toc164460123)

[Supporting Tables 6](#_Toc164460124)

[Supporting Figures 8](#_Toc164460125)

[Supporting References 42](#_Toc164460126)

**Supporting Note S1**

#### *Quantitation Details*

Several DNA regions were amplified simultaneously using different primers with different probes. Each primer targets specific DNA regions to provide different information ^[1]^, as summarized below.

[*A*] is the concentration of total human DNA in pg µL^−1^ or ng µL^−1^. This is multiplied by 50 µL (the eluate volume) to calculate the absolute amount *A* (ng).

[*Y*] is the concentration of male human DNA in pg µL^−1^ or ng µL^−1^. This is multiplied by 50 µL (the eluate volume) to calculate the absolute amount *Y* (ng). [*A*]/[*Y*] or *A*/*Y* is an indicator of male DNA purity and is used to assess the purity of the sperm fraction.

[*D*] is the concentration of total human DNA in pg µL^−1^ or ng µL^−1^, measured using a longer sequence (294 bp) than that used for [*A*] (84 bp). Because it is a longer region, it is more susceptible to degradation. This is multiplied by 50 µL (the eluate volume) to calculate the absolute amount *D* (ng). The ratio [*A*]/[*D*] or *A*/*D* is an indicator of DNA degradation.

#### *Male DNA Extraction Efficiency*

The primers used by PowerQuant® to quantify male DNA target two multicopy loci in the Y chromosome, one locus is an 81 bp amplicon, and the other is a 136 bp amplicon.^[1]^ The qPCR reaction measures the concentration of total male DNA, [*Y*], in ng µL^−1^.

The male DNA extraction efficiency, EE, was calculated using equation (1),

|  | $\text{EE}= \frac{\left[ Y \right]\times V_{elution}}{S\times Y_{single sperm}}=\frac{Y_{extracted}}{Y_{total}}\times100\%$ | (1) |
| --- | --- | --- |

where $Y_{extracted}$ is the amount of DNA extracted in ng, calculated by multiplying [*Y*] by $V_{elution}$ the eluate volume, $Y_{total}$ is the amount of DNA added to the swab in ng, calculated by multiplying $S$, the number of sperm cells added to the swab, and $Y_{single sperm}$, an estimate of the amount of DNA per sperm cell, 3.3 pg.^[2]^ $S$ was calculated by multiplying sperm density for each of the 1:10 and 1:100 dilutions (as determined using a hemacytometer) with the volume of diluted semen added to the swab.

#### *Male DNA Purity*

The qPCR reaction measures the concentration of male human DNA, [*Y*]. Theoretically, samples with only male DNA should have a [*A*]/[*Y*] ratio of 1.0. It was found that this ratio can vary from as low as 0.53 up to 1.83 for PowerQuant® ^[1]^ even for samples that are known to consist of purely male DNA. The manufacturer suggests to use a range of 0.5 - 2 as evidence of a sample contains primarily male DNA.^[1]^ This is an improvement over PlexorHY®, where purely male DNA can have [*A*]/[*Y*] up to 4.2.^[3]^

#### *DNA degradation*

The third amplicon amplified by PowerQuant® is a long (294 bp) DNA region that is more susceptible to degradation. The manufacturer suggests using a threshold of [*A*]/[*D*] > 2 to indicate that a sample is degraded.

**Supporting Note S2**

#### *Chemometric Optimization*

We explored the use of chemometrics^[4]^ to optimize the DMF-DD protocol for efficiency and processing time. In this study, Plackett-Burman analysis was used to determine the parameters with statistically significant effects on the variable being optimized, followed by Yates Pattern analysis to determine how to regulate the parameters simultaneously to achieve optimization.

Plackett-Burman analysis consists of conducting trials that test each parameter at low and high extremes.^[5]^ We used the technique to evaluate six parameters in the DMF-DD protocol: the concentration of DNase, the concentrations of its cofactors Ca^2+^ and Mg^2+^, DNase incubation time, EDTA incubation time, and DTT incubation time by testing each parameter at its low and high extremes as outlined in Table S2. To account for confounding variables, the test also evaluated “unassigned” parameters outside six parameters of interest, designed to mimic the effect of noise contributions to the results.^[6]^

Prior to automating the DD method on a DMF platform, the method was first optimized in tubes. Twelve optimization trials were performed; the chemometric permutations of each trial are outlined in Table S2. For each trial, three biological replicates [i.e., three buccal swabs containing cheek cells from a female volunteer spiked with 50 µL diluted semen (1:10)] were used, with each sample tested three times (technical replicates).

To execute these trials, low and high concentrations of MgCl_2_ (BDH Inc.) and CaCl_2_ (BDH Inc.) were prepared in 100 mm tris-HCl (pH 7.5 at 20℃), which was prepared using 100 mm Trizma^®^ base (Sigma) in 1 m HCl. Separately, low and high concentrations of DNase solutions were prepared by dissolving DNase I lyophilized powder (D5025, Sigma-Aldrich) in 50 mm tris-HCl (pH 7.5 at 20℃), 10 mm CaCl_2_, and 50% (v/v) glycerol.

Reasonable and appropriate low and high extreme values informed by literature were set for each parameter. For the DNase concentration, the low was set to 0.5 Kunitz µL^−1^ and the high was set to 20 Kunitz µL^−1^.^[7]^ For the cofactor cations, [Ca^2+^] and [Mg^2+^] were set to 0.5 mm (low) and 10 mm (high), and 5 mm (low) and 25 mm (high), respectively.^[7,8]^ For the incubation times of DNase, EDTA, and DTT, the protocol outlined by Wong et al. included 15, 10, and 15 minutes respectively.^[8]^ To minimize the incubation time while also allowing enough time for the reactions to proceed, the low times were set to 1 minute, and the high times were set to that of the protocol outlined by Wong et al.^[8]^ PCR analysis to determine [*A*]/[*Y*] was determined using PowerQuant® (Promega) kits as described in the main text and in Supporting Note 1. A Plackett-Burman analysis identified that at a 70% confidence interval, DNase and EDTA incubation times exceeded the minimum effect threshold, thus were deemed to be statistically significant and were further optimized through Yates Pattern Analysis.

Four Yates Pattern analysis^[5]^ trials (each with three biological replicates) were performed to evaluate DNase and EDTA incubation times. The experimental data were fit to polynomial equations with format *y = a + bχ1 + cχ2 + dχ1χ2*, where *y* is the variable being optimized (in this case, [*A*]/[*Y*] ratio), *a* is the mean effect, *χ1* and *χ2* are the significant parameters (DNase and EDT incubation times), *χ1χ2* is the product of the significant parameters, and *b*, *c*, and *d* are the effects of *χ1*, *χ2*, and *χ1χ2*, respectively. The polynomial equation allows the experimenter to input the values of *χ1* and *χ2* that yield an optimal value for *y*, without needing to perform an actual experiment, thereby saving time and resources.

The results of the Yates pattern analysis are given in Table S3. As shown, neither DNase nor EDTA incubation times were found to be statistically significant; that is, the resulting equation was *y*= 0.93389, indicating that *y* is a mean effect. Note that the result of *y*= 0.93389 as opposed to *y*= 0 means that the two statistically significant parameters according to Plackett-Burman do interact; however, the interaction does not have a tangible effect on the measured results. In future experiments, a broader range of values, such as comparing 1 minute to 60 minutes, might reveal a more statistically significant effect.

Regardless of the Yates pattern analysis, the results generated here suggested a substantial reduction in processing time could be achieved for DMF-DD processing of mixed DNA samples. To test this hypothesis, the protocol for the optimized trial with the lowest incubation times (Trial 4) was selected to be performed on DMF as a proof-of-concept as it consisted of the lowest incubation time (3 minutes) while producing [*A*]/[*Y*] less than 2 (1.25 ± 0.22). To perform this method on DMF, the volumes in the protocol were scaled to be appropriate for the DMF method (keeping concentrations, times, and other conditions constant). The protocol was then programmed in MicroDrop (with a total run time of approx. 5 minutes) to operate on the Zed-box.

Figure S36 report the [*A*]/[*Y*] measured for the benchtop method carried out according to the standard method, the Trial 4 method conducted in tubes with scaled-down volumes (appropriate for DMF), and the Trial 4 method conducted on DMF. As shown, the [A]/[Y] values generated using the Trial 4 method (3 minutes incubation) are not statistically distinguishable from those generated using the standard method (45 minutes incubation). Specifically, a two-sample t-test indicated there is no statistically significant difference between the [*A*]/[*Y*] of the standard method and the Trial 4 method in tubes at 95% confidence interval (*t* = 0.42, *p* = 0.70). And the same test indicated no difference between the standard method and the DMF technique at 95% confidence interval (*t =* 1.11, *p =* 0.33 and *t =* 1.57*, p =* 0.19 respectively). This result is remarkable and while it has not yet been applied to post-coital sample analysis or STR genotyping, it bodes well for potential reductions in sample processing time for this application.

# **Supporting Video**

Supporting Video S1.

Description: Demonstration of the steps performed using the DMF chip. For this demonstration, the liquids moved were PBS with food dyes added for visualization.

# **Supporting Tables**

Table S1. DNA quantitation of sperm (SF) and non-sperm (NSF) fractions from samples collected 1-72 hours post-coitus (PC). Quantitation was performed using PowerQuant® qPCR kit, with concentration (for [*A*], [*Y*] and [*D*]) reported in ng µL^−1^ [mean of 2 technical replicates per condition (SD)]. NSF samples were processed by manual DD only; SF samples were processed using DMF-DD, apart from SF*, which was processed manually.

| Sample | Time PC | Fraction | [*A*] | [*Y*] | [*D*] | [*A*]/[*D*] | [*A*]/[*Y*] |
| --- | --- | --- | --- | --- | --- | --- | --- |
| 1 | **1 hr** | **SF*** | 11.5 (6.4) | 7.4 (3.8) | 12.3 (5.3) | 0.9 (0.13) | 1.6 (0.07) |
|  |  | **SF** | 7.1 (4.0) | 3 (0.19) | 5.6 (0.80) | 1.3 (0.53) | 2.3 (1.16) |
|  |  | **NSF** | 63.3 (3.0) | 0.41 (0.01) | 42.5 (0.42) | 1.5 (0.08) | 153.9 (12.6) |
| 2 | **3 hr** | **SF** | 22 (1.4) | 21.5 (1.42) | 21.4 (0.36) | 1.0 (0.08) | 1.0 (0.007) |
|  |  | **NSF** | 2.1 (0.14) | 0.07 (0.005) | 1.3 (0.12) | 1.6 (0.04) | 29.1 (0.06) |
| 3 | **6 hr** | **SF** | 0.16 (0.001) | 0.16 (0.006) | 0.15 (0.005) | 1.1 (0.03) | 1.0 (0.05) |
|  |  | **NSF** | 6 (0.18) | 0.01 (0.002) | 5.1 (0.57) | 1.2 (0.099) | 508.0 (54.9) |
| 4 | **1 hr** | **SF** | 3.0 (0.14) | 2.6 (0.08) | 3.0 (0.1) | 1.0 (0.01) | 1.1 (0.02) |
|  |  | **NSF** | 18.6 (0.85) | 0.23 (0.009) | 12.8 (0.98) | 1.5 (0.05) | 79.6 (0.64) |
| 5 | **3 hr** | **SF** | 2.0 (0.09) | 1.8 (0.03) | 2.3 (0.05) | 0.9 (0.06) | 1.1 (0.04) |
|  |  | **NSF** | 12.9 (0.54) | 0.099 (0.002) | 4.09 (0.009) | 3.2 (0.13) | 130.2 (3.1) |
| 6 | **6 hr** | **SF** | 1.2 (0.07) | 1.2 (0.07) | 1.6 (0.05) | 0.8 (0.07) | 1.0 (0.12) |
|  |  | **NSF** | 18.4 (6.9) | 0.07 (0.01) | 5.6 (1.35) | 3.3 (0.45) | 280.1 (50.8) |
| 7 | **12 hr** | **SF** | 1.1 (0.08) | 0.8 (0.03) | 1.2 (0.07) | 0.9 (0.01) | 1.3 (0.05) |
|  |  | **NSF** | 2.6 | 0.02 | 0.86 | 3.0 | 126.6 |
| 8 | **24 hr** | **SF** | 1.7 (0.09) | 0.48 (0.008) | 1.1 (0.02) | 1.5 (0.11) | 3.5 (0.13) |
|  |  | **NSF** | 2.2 (0.03) | 0.03 (0.003) | 1.4 (0.05) | 1.6 (0.03) | 74 (8.2) |
| 9 | **24 hr** | **SF** | 1.2 (0.28) | 0.67 (0.06) | 1.0 (0.01) | 1.2 (0.25) | 1.9 (0.58) |
|  |  | **NSF** | 1.3 (0.22) | 0.006 (0.0004) | 0.97 (0.13) | 1.3 (0.04) | 235 (25) |
| 10 | **48 hr** | **SF** | 0.55 (0.20) | 0.71 (0.09) | 1.12 (0.19) | 0.49 (0.26) | 0.77 (0.38) |
|  |  | **NSF** | 58.04 (7.2) | 0.019 (0.002) | 52.72 (6.4) | 1.1 (0.002) | 3007 (63) |
| 11 | **72 hr** | **SF** | 6.5 (0.33) | 0.56 (0.1) | 5.47 (0.8) | 1.2 (0.2) | 11.5 (2.7) |
|  |  | **NSF** | 93.8 (22.3) | 0.013 (0.002) | 79.8 (26.6) | 1.2 (0.12) | 7103 (670) |

**Table S2.** **Plackett-Burman analysis parameters.** Conditions for 12 trials measuring [*A*]/[*Y*] for six variables by PCR analysis (PowerQuant®, Promega) for *n* = 3 biological replicates of sperm fractions extracted from buccal swabs from a female volunteer spiked with 50 µL semen diluted 1:10. Lows and highs for the six variables are shown in red and blue, respectively. Trial 4 is highlighted in green because it was the shortest trial with A/Y <2.

| **Trial** | **[Ca^2+^]** | **[Mg^2+^]** | **[Dnase]** | **Dnase *t*** | **EDTA *t*** | **DTT *t*** | **[*A*]/[*Y*] (s.d.)** |
| --- | --- | --- | --- | --- | --- | --- | --- |
| **1** | 10 mM | 25 mM | 0.5 Kunitz/µL | 15 min | 10 min | 15 min | 1.2 (0.1) |
| **2** | 10 mM | 5 mM | 20 Kunitz/µL | 15 min | 10 min | 1 min | 1.7 (1.0) |
| **3** | 0.5 mM | 25 mM | 20 Kunitz/µL | 15 min | 1 min | 1 min | 3.0 (1.1) |
| **4** | 10 mM | 25 mM | 20 Kunitz/µL | 1 min | 1 min | 1 min | **1.3 (0.2)** |
| **5** | 10 mM | 25 mM | 0.5 Kunitz/µL | 1 min | 1 min | 15 min | 3.0 (2.0) |
| **6** | 10 mM | 5 mM | 0.5 Kunitz/µL | 1 min | 10 min | 1 min | 1.6 (0.4) |
| **7** | 0.5 mM | 5 mM | 0.5 Kunitz/µL | 15 min | 1 min | 15 min | 1.7 (0.5) |
| **8** | 0.5 mM | 5 mM | 20 Kunitz/µL | 1 min | 10 min | 15 min | 1.4 (0.3) |
| **9** | 0.5 mM | 25 mM | 0.5 Kunitz/µL | 15 min | 10 min | 1 min | 1.1 (0.3) |
| **10** | 10 mM | 5 mM | 20 Kunitz/µL | 15 min | 1 min | 15 min | 1.2 (0.5) |
| **11** | 0.5 mM | 25 mM | 20 Kunitz/µL | 1 min | 10 min | 15 min | 1.3 (0.6) |
| **12** | 0.5 mM | 5 mM | 0.5 Kunitz/µL | 1 min | 1 min | 1 min | 3.4 (3.2) |

**Table S3. Yates pattern analysis parameters.** Conditions for 4 trials (13-17) measuring [*A*]/[*Y*] for two variables (𝛘**_1_** - DNase incubation time and 𝛘**_2_** - EDTA incubation time) by PCR analysis (PowerQuant®, Promega) for *n* = 3 biological replicates of sperm fractions extracted from buccal swabs from a female volunteer spiked with 50 µL semen diluted 1:10. Trial 18 is the midpoint trial.

| Trial | Repeats | $\boldsymbol{\chi}_{\boldsymbol{1}}$(DNase incubation time) | $\boldsymbol{\chi}_{\boldsymbol{2}}$(EDTA incubation time) | [*A*]/[*Y*] (s.d.) |
| --- | --- | --- | --- | --- |
| **13** | 3 | 1 min | 1 min | 0.97 (0.20) |
| **14** | 3 | 15 min | 1 min | 0.92 (0.25) |
| **15** | 3 | 1 min | 10 min | 0.88 (0.17) |
| **17** | 3 | 15 min | 10 min | 0.95 (0.38) |
| **18** | 5 | 8 min | 5.5 min | 0.91 (0.35) |

# **Supporting Figures**

| *(a) Manual Differential Digestion (DD) protocol* | *(b) DMF-assisted Differential Digestion (DMF-DD)* |
| --- | --- |

Figure S1. Comparison of the manual differential digestion protocol (DD) and DMF-Assisted differential digestion (DMF-DD).

Schematics illustrating the steps required in (a) manual DD and (b) DMF-DD. (M) and orange color indicates that the step is performed manually, (A) and green color indicates that the step is performed automatically. As shown, in DMF-DD the operator carries out 5 steps manually (1 to 5), and the DMF instrument performs the remaining 8 steps (i to viii).


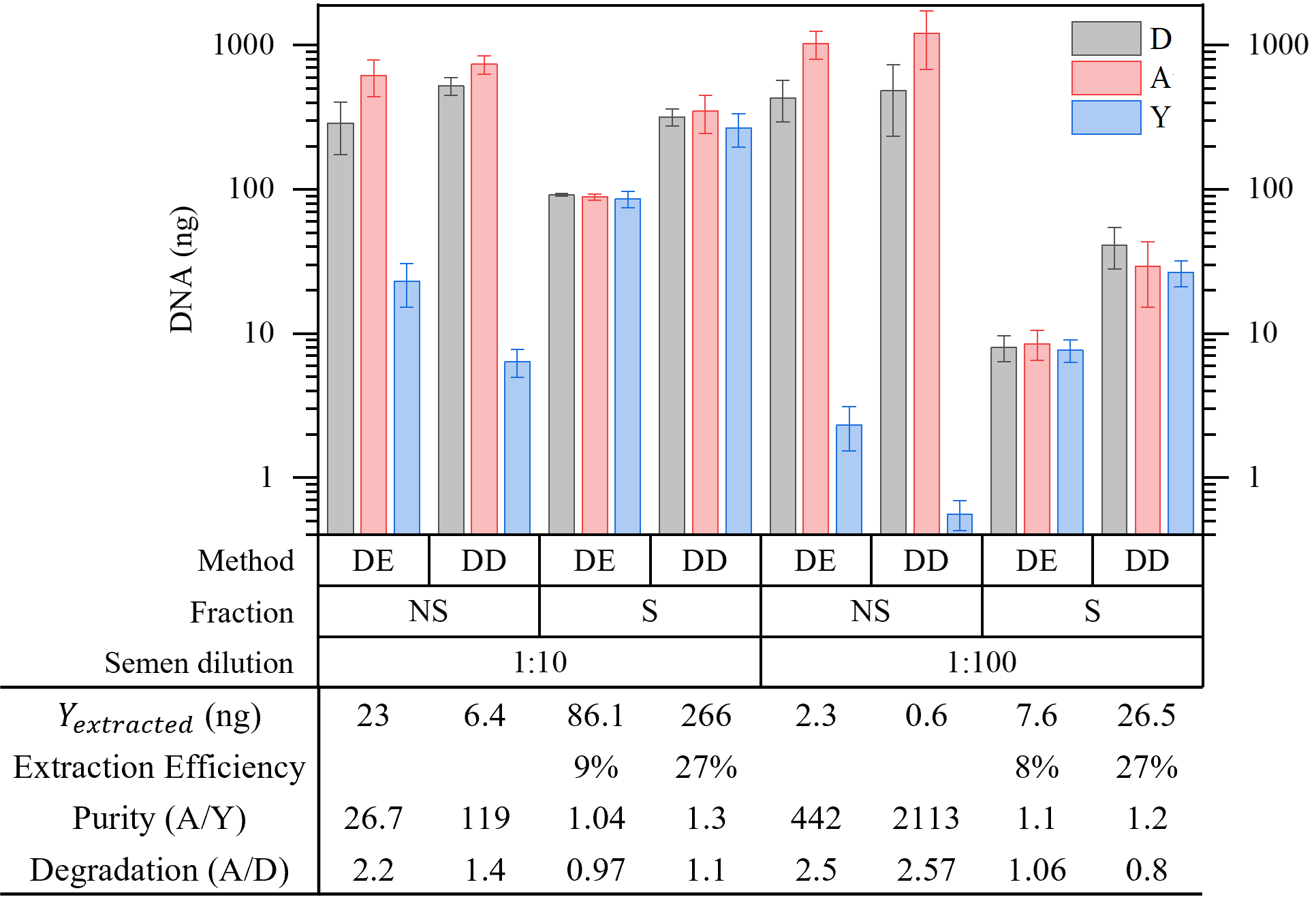


Figure S2. Comparison of differential extraction (DE) and differential digestion (DD).

qPCR results showing the amount of DNA in nanograms (ng) recovered in non-sperm (NS) and sperm (S) fractions from buccal swabs spiked with semen standards diluted at 1:10 or 1:100 ratios, processed manually using either differential extraction (DE) or differential digestion (DD). Grey bars represent the amount of autosomal DNA measured using a long amplicon susceptible to degradation, D. Red bars represent the amount of autosomal DNA measured using a short amplicon, A. Blue bars represent the amount of male DNA, Y. Four measurements were collected for each condition, comprising 2 biological replicates (2 different swabs) and two technical replicates (2 different qPCR reactions per biological replicate). The reported values represent the means of the 4 measurements, and error bars are ± 1 standard deviation.


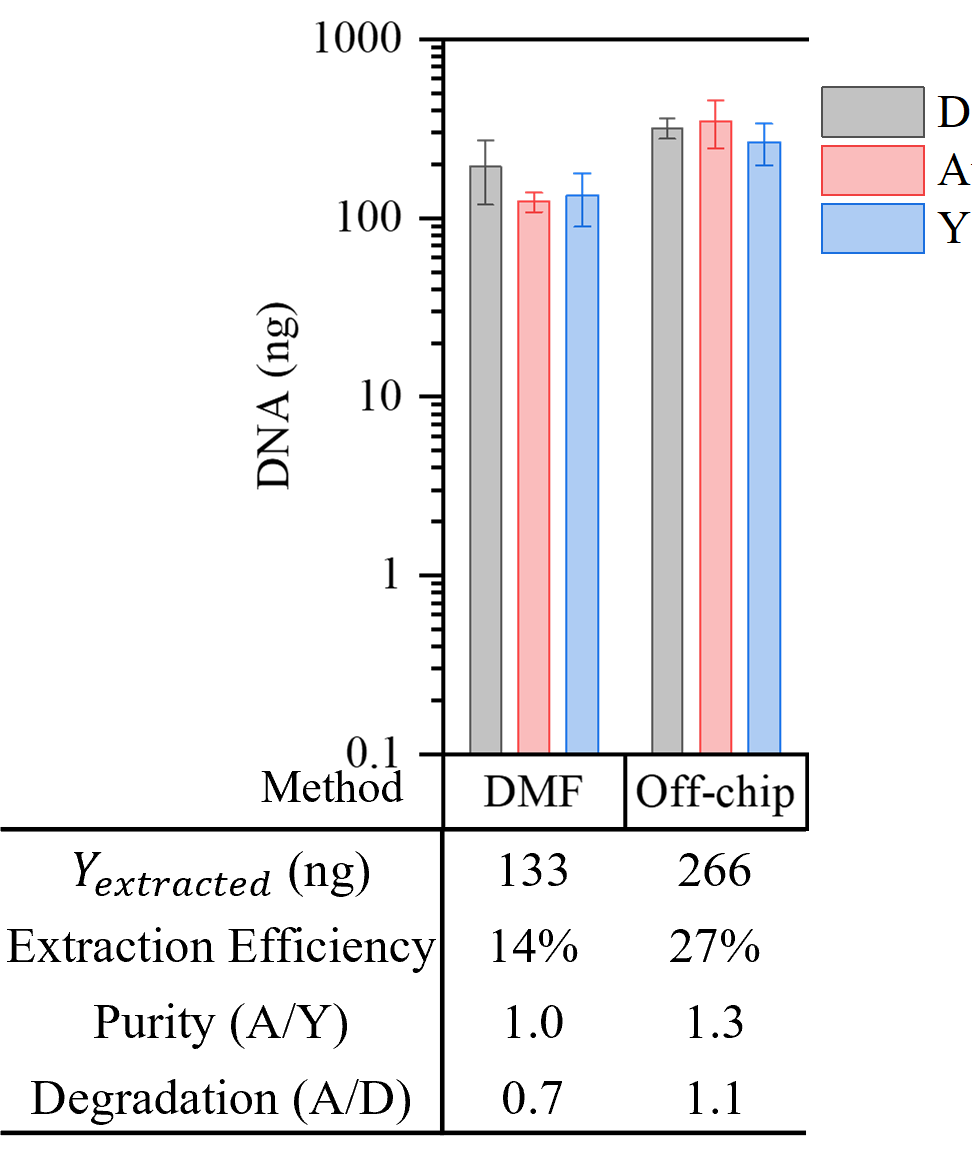


Figure S3. Comparison of DMF-DD to manual DD (“off-chip”).

qPCR results showing the amount of DNA in nanograms (ng) recovered in the sperm fraction from buccal swabs spiked with semen diluted at 1:10. Grey bars represent the amount of autosomal DNA measured using a long amplicon susceptible to degradation, *D*. Red bars represent the amount of autosomal DNA measured using a short amplicon, *A*. Blue bars represent the amount of male DNA, *Y*. Two measurements were collected for each condition. The reported values represent the mean of the measurements, and error bars are ± 1 standard deviation.


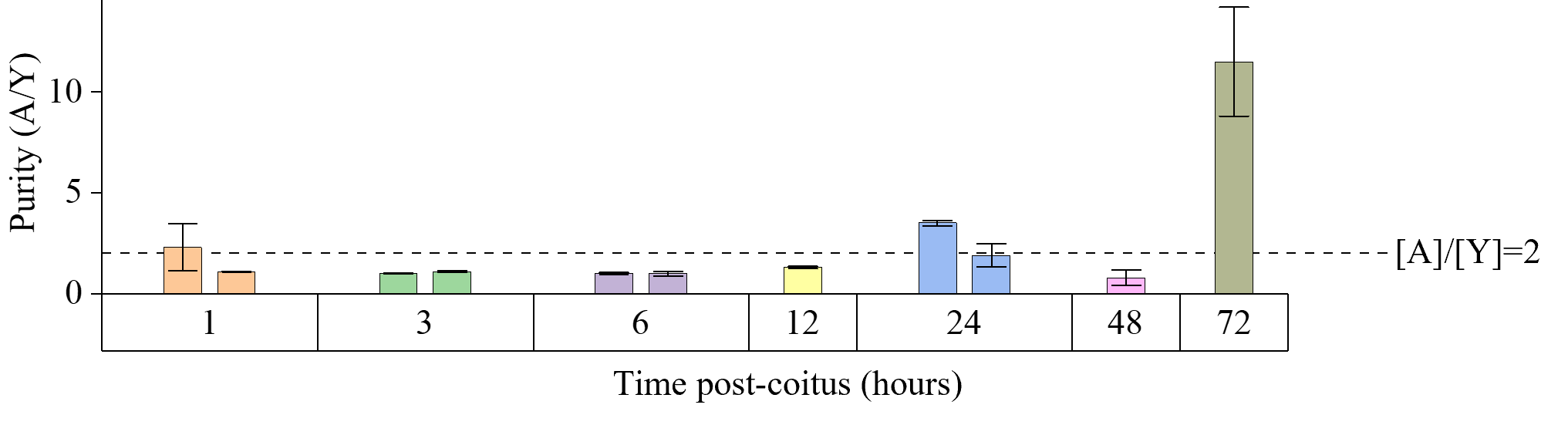


Figure S4. Purity of Post-Coital Samples. Plot of [A]/[Y] (ratio of concentrations of an autologous locus found in all DNA to a locus only found on the male chromosome) for samples 1 and 4 (orange), 2 and 5 (light green), 3 and 6 (purple), 7 (yellow), 8 and 9 (blue), 10 (pink), and 11 (dark green), that were collected 1, 3, 6, 12, 24, 48, or 72 hours PC and processed by DMF-DD. The bars report the mean of two technical replicates per sample, and error bars are ± 1 standard deviation. The dashed lined denotes the cutoff between “single” (male) and “mixed” (male/female) sources, [*A*]/[*Y*] = 2.

Sample 1 – 1 hr post-coitus – NSF electropherogram


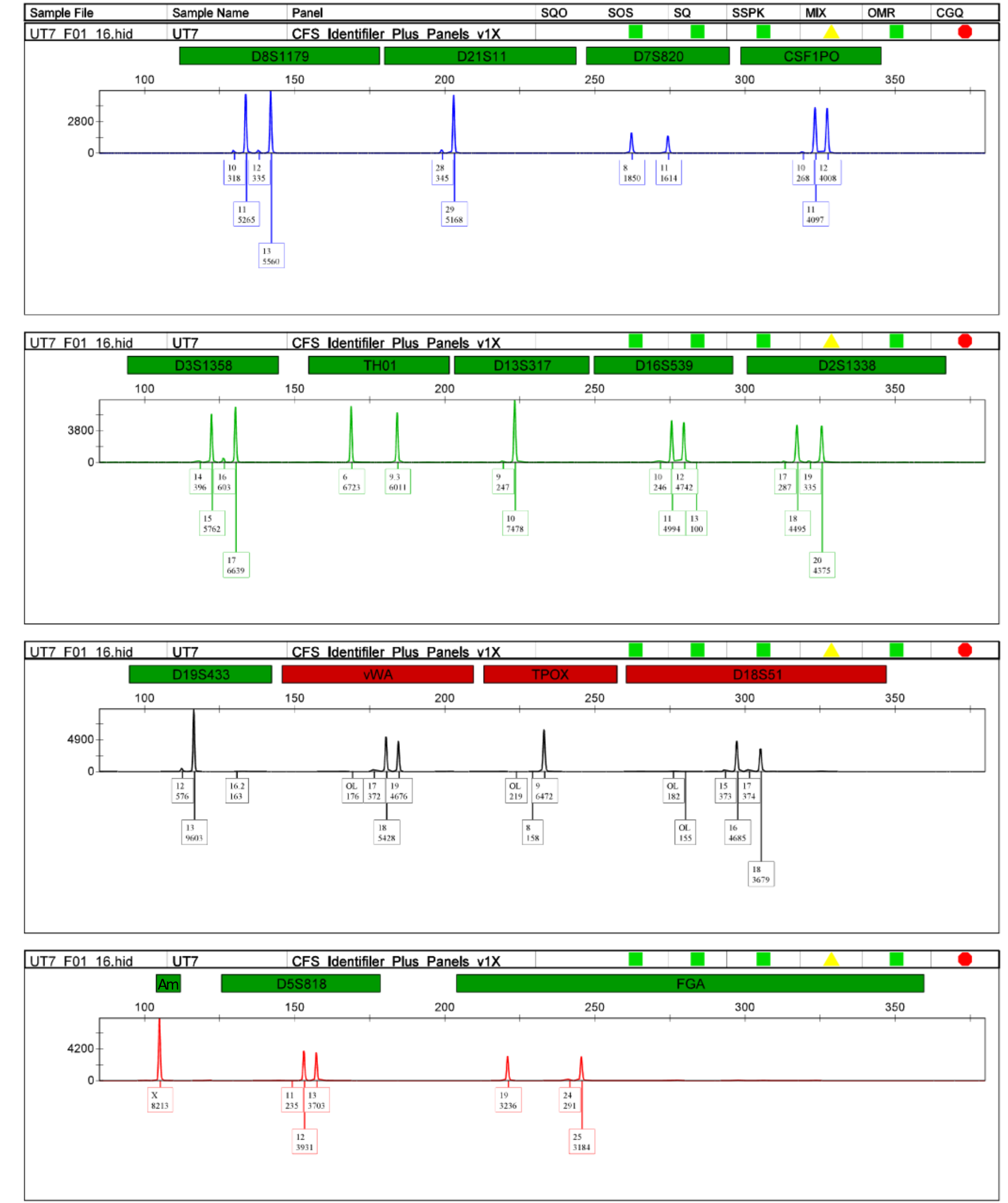


Figure S5. Electrophoretic analysis of NSF for sample 1 generated using standard laboratory analysis techniques.

Data are shown as traces of fluorescence intensity for each channel (blue, green, black, or red) as a function of fragment length (bp). In each panel, labels above the plot (green and red filled boxes) indicate which markers the peaks correspond to, including the sex-typing marker Amelogenin (“Am”) and STR loci D8S1179, D21S11, D7S820, CSF1PO, D3S1358, TH01, D13S317, D16S539, D2S1338, D19S433, vWA, TPOX, D18S51, D5S818, and FGA. Labels below the plot (blue, green, black, and red outlined boxes) indicate either the number of repeats for STR loci or X / Y designation for sex typing, as determined using GeneMapper® ID-X (Applied Biosystems), as well as the peak height.

Sample 1 – 1 hr post-coitus – SF*, tubes, lab-based DNA analysis, electropherogram


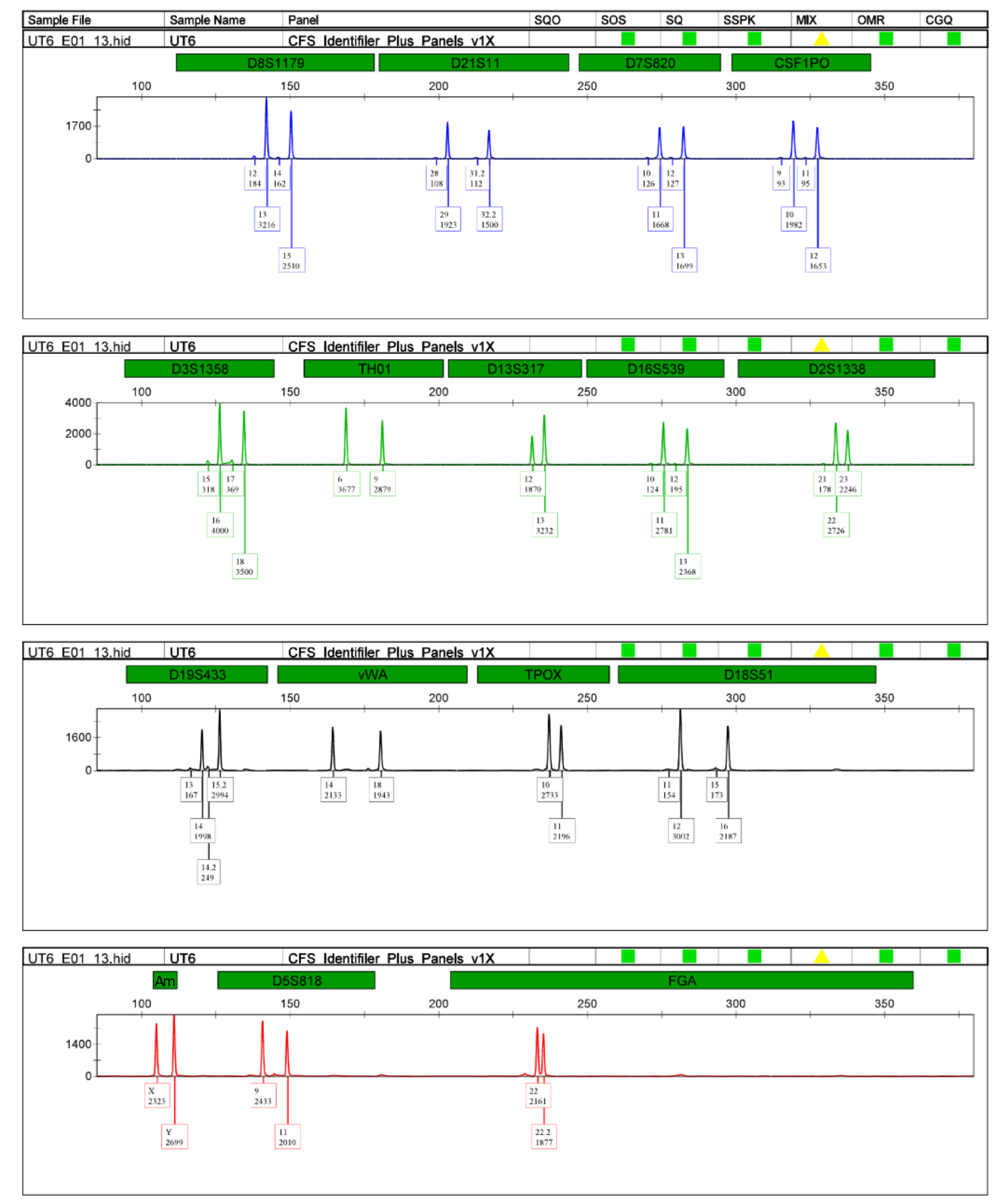


Figure S6. Electrophoretic analysis of SF for sample 1, processed using DD in tubes, generated using standard laboratory analysis techniques.

Data are shown as traces of fluorescence intensity for each channel (blue, green, black, or red) as a function of fragment length (bp). In each panel, labels above the plot (green boxes) indicate which markers the peaks correspond to, including the sex-typing marker Amelogenin (“Am”) and STR loci D8S1179, D21S11, etc. Labels below the plot (blue, green, black, and red outlined boxes) indicate either the number of repeats for STR loci or X / Y designation for sex typing, as determined using GeneMapper® ID-X (Applied Biosystems), as well as the peak height.

Sample 1 – 1 hr post-coitus – SF, DMF, lab-based DNA analysis, electropherogram


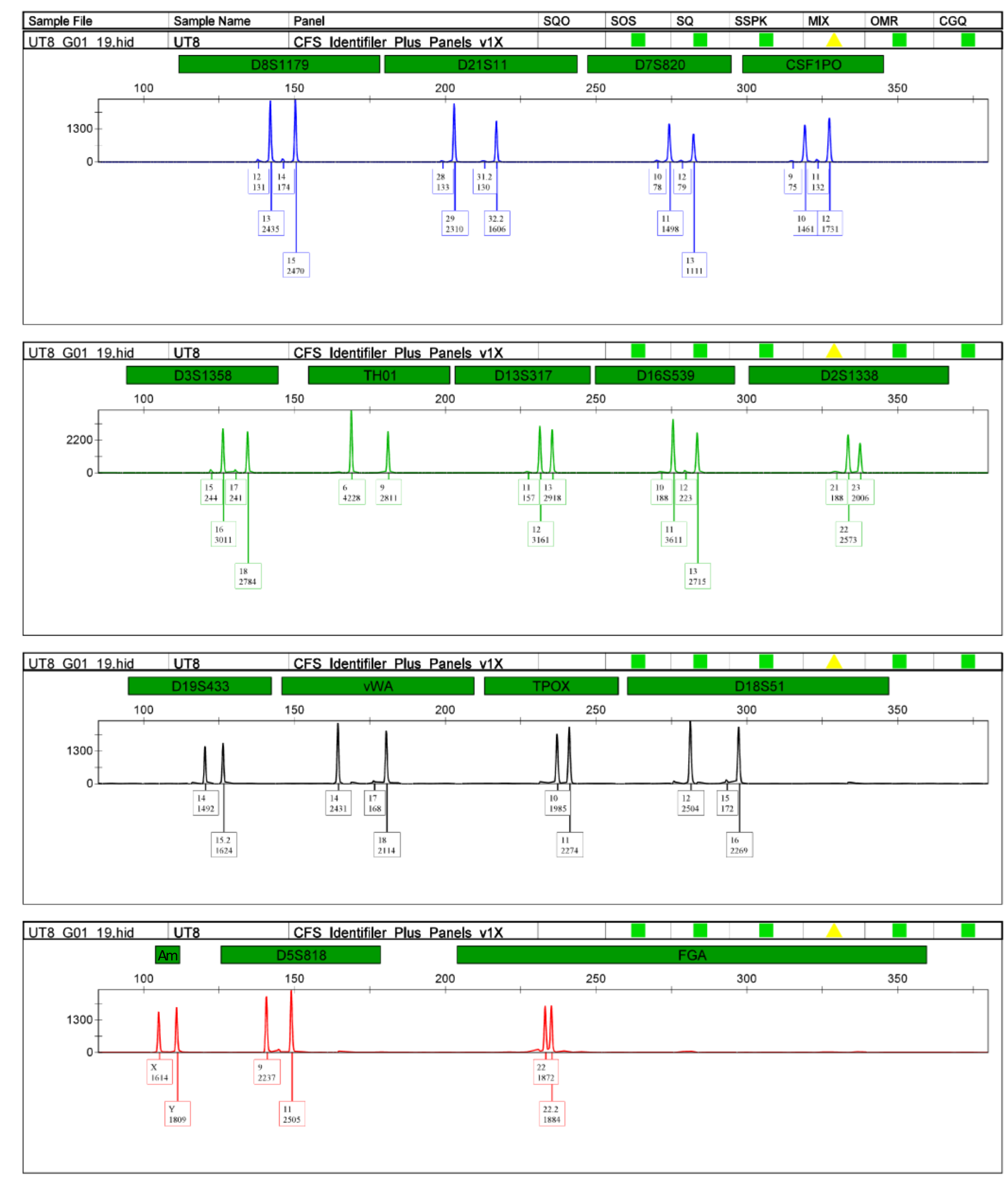


Figure S7. Electrophoretic analysis of SF for sample 1, processed using DMF-DD, generated using standard laboratory analysis techniques.

Data are shown as traces of fluorescence intensity for each label (blue, green, black, or red) as a function of fragment length (bp). In each panel, labels above the plot (green and red filled boxes) indicate which markers the peaks correspond to, including the sex-typing marker Amelogenin (“Am”) and STR loci D8S1179, D21S11, etc. Labels below the plot (blue, green, black, and red outlined boxes) indicate either the number of repeats for STR loci or X / Y designation for sex typing, as determined using GeneMapper® ID-X (Applied Biosystems), as well as the peak height.

Sample 2 – 3 hrs post-coitus – NSF electropherogram


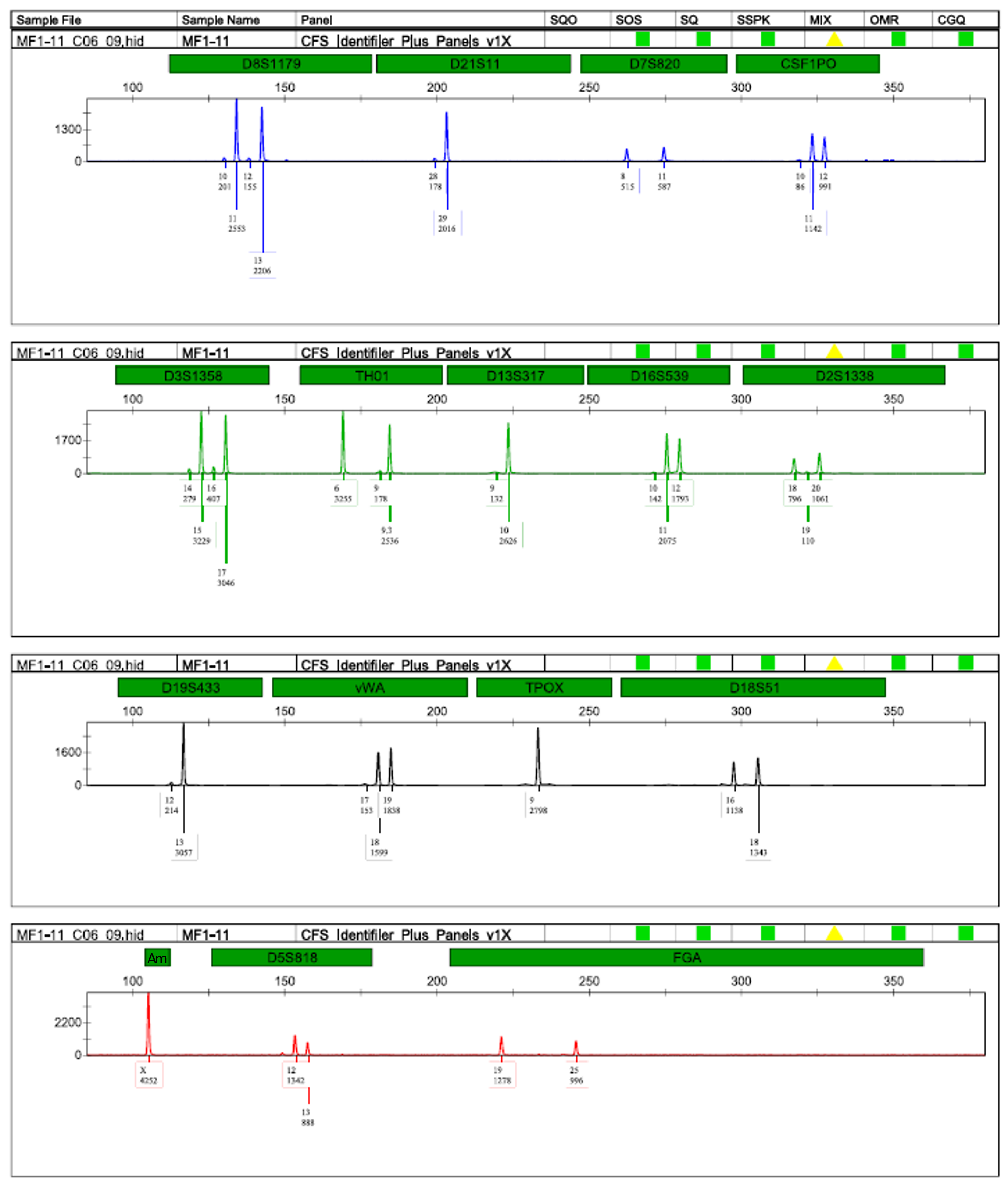


Figure S8. Electrophoretic analysis of NSF for sample 2 generated using standard laboratory analysis techniques.

Data are shown as traces of fluorescence intensity for each channel (blue, green, black, or red) as a function of fragment length (bp). In each panel, labels above the plot (green filled boxes) indicate which markers the peaks correspond to, including the sex-typing marker Amelogenin (“Am”) and STR loci D8S1179, D21S11, etc. Labels below the plot (blue, green, black, and red outlined boxes) indicate either the number of repeats for STR loci or X / Y designation for sex typing, as determined using GeneMapper® ID-X (Applied Biosystems), as well as the peak height.

Sample 2 – 3 hrs post-coitus – SF, DMF, lab-based DNA analysis, electropherogram


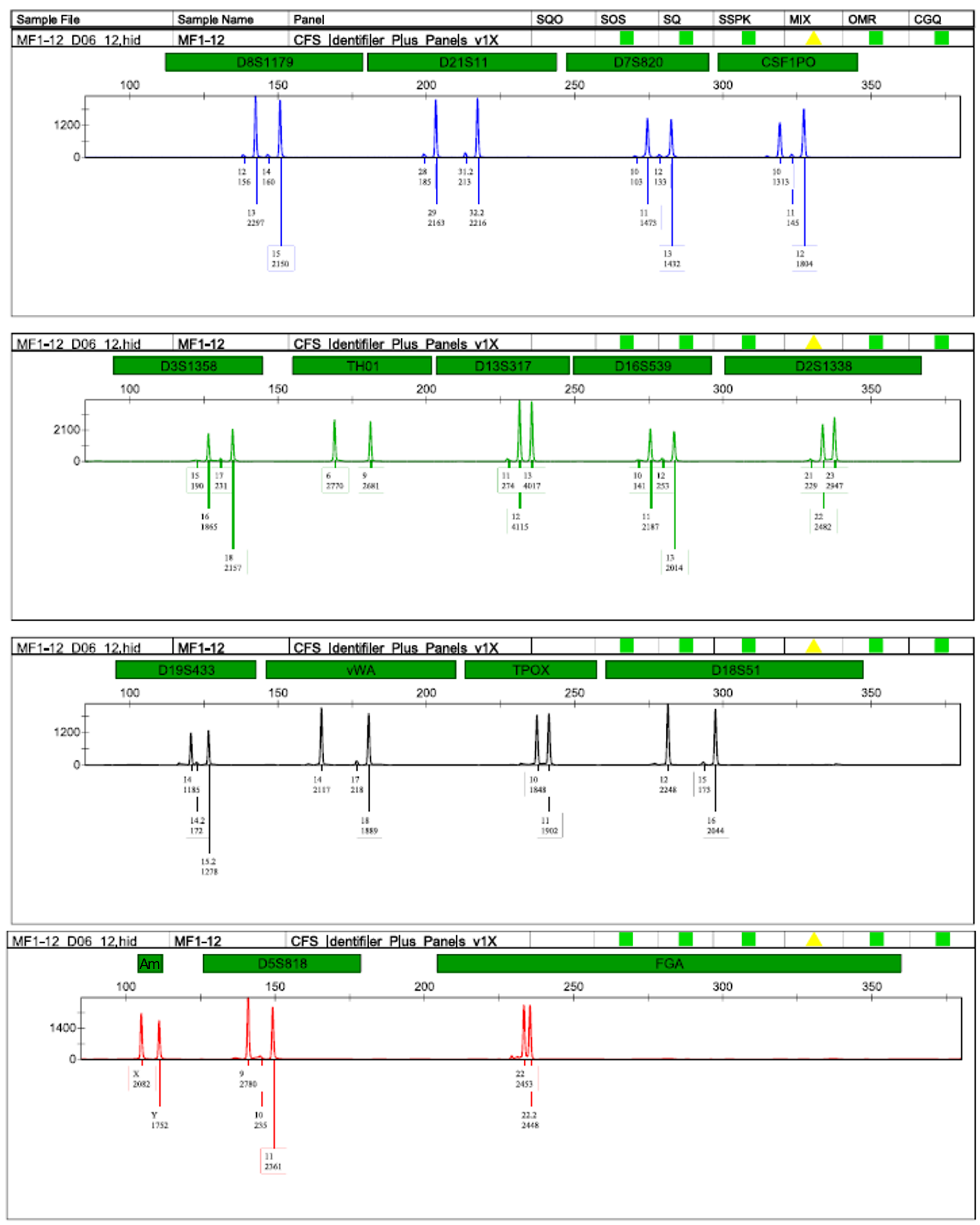


Figure S9. Electrophoretic analysis of SF for sample 2, processed using DMF-DD, generated using standard laboratory analysis techniques.

Data are shown as traces of fluorescence intensity for each channel (blue, green, black, or red) as a function of fragment length (bp). In each panel, labels above the plot (green filled boxes) indicate which markers the peaks correspond to, including the sex-typing marker Amelogenin (“Am” in the plot) and STR loci D8S1179, D21S11, etc. Labels below the plot (blue, green, black, and red outlined boxes) indicate either the number of repeats for STR loci or X / Y designation for sex typing, as determined using GeneMapper® ID-X (Applied Biosystems), as well as the peak height.

Sample 3 – 6 hrs post-coitus – NSF electropherogram


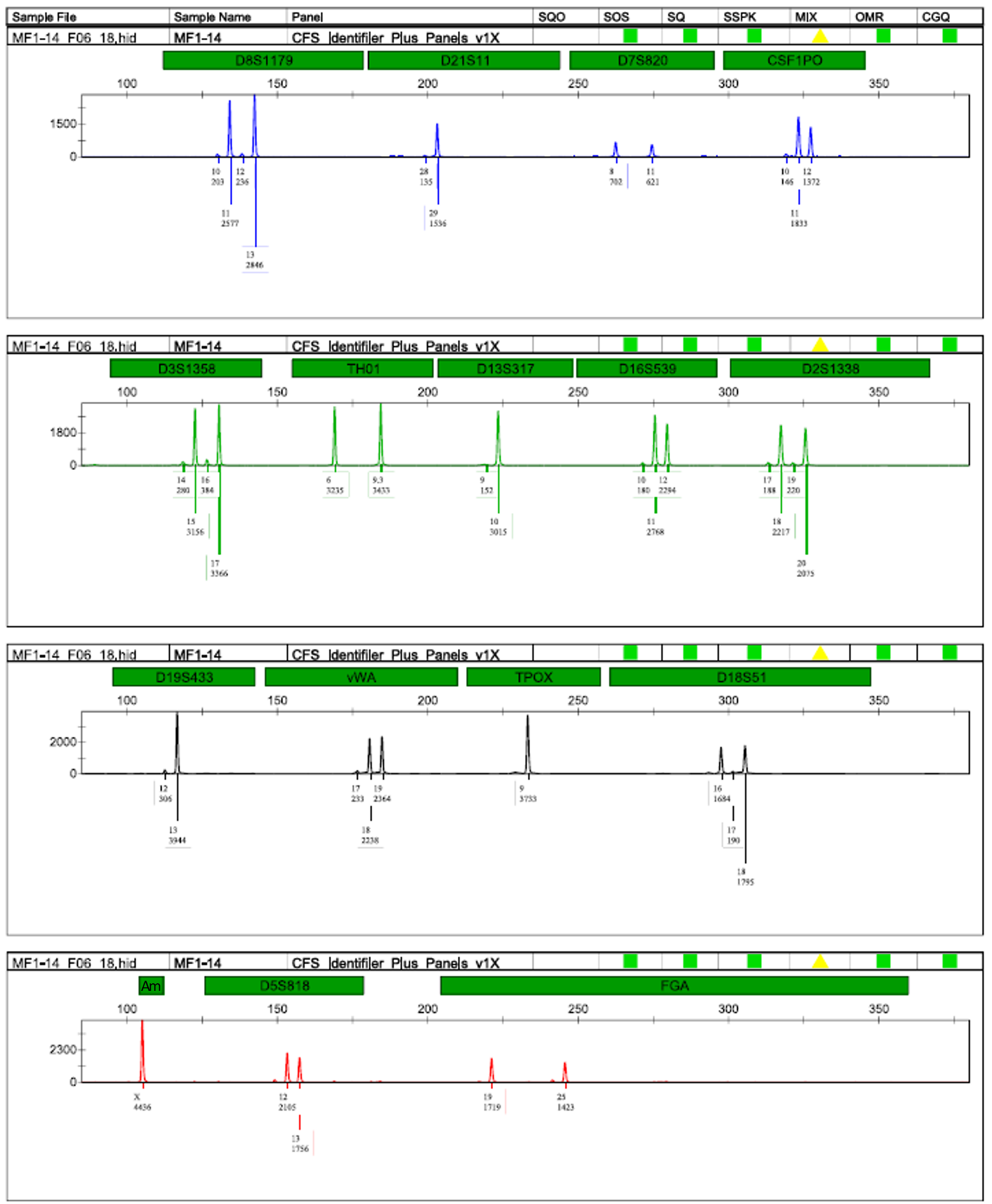


Figure S10. Electrophoretic analysis of NSF for sample 3 generated using standard laboratory analysis techniques.

Data are shown as traces of fluorescence intensity for each channel (blue, green, black, or red) as a function of fragment length (bp). In each panel, labels above the plot (green filled boxes) indicate which markers the peaks correspond to, including the sex-typing marker Amelogenin (“Am”) and STR loci D8S1179, D21S11, etc. Labels below the plot (blue, green, black, and red outlined boxes) indicate either the number of repeats for STR loci or X / Y designation for sex typing, as determined using GeneMapper® ID-X (Applied Biosystems), as well as the peak height.

Sample 3 – 6 hrs post-coitus – SF, DMF, lab-based DNA analysis, electropherogram


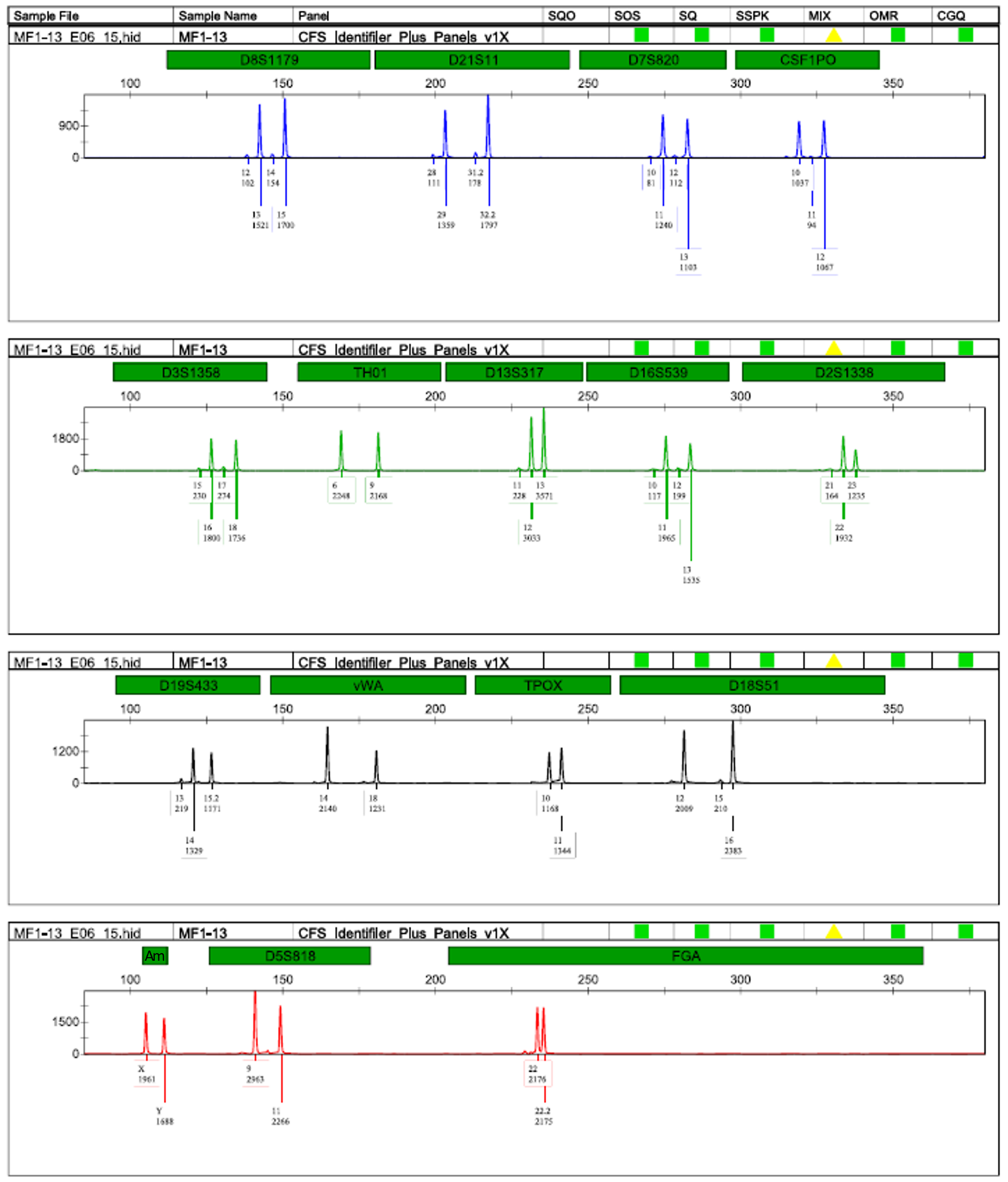


Figure S11. Electrophoretic analysis of SF for sample 3, processed using DMF-DD, generated using standard laboratory analysis techniques.

Data are shown as traces of fluorescence intensity for each channel (blue, green, black, or red) as a function of fragment length (bp). In each panel, labels above the plot (green filled boxes) indicate which markers the peaks correspond to, including the sex-typing marker Amelogenin (“Am”) and STR loci D8S1179, D21S11, etc. Labels below the plot (blue, green, black, and red outlined boxes) indicate either the number of repeats for STR loci or X / Y designation for sex typing, as determined using GeneMapper® ID-X (Applied Biosystems), as well as the peak height.

Sample 4 – 1 hr post-coitus – NSF electropherogram


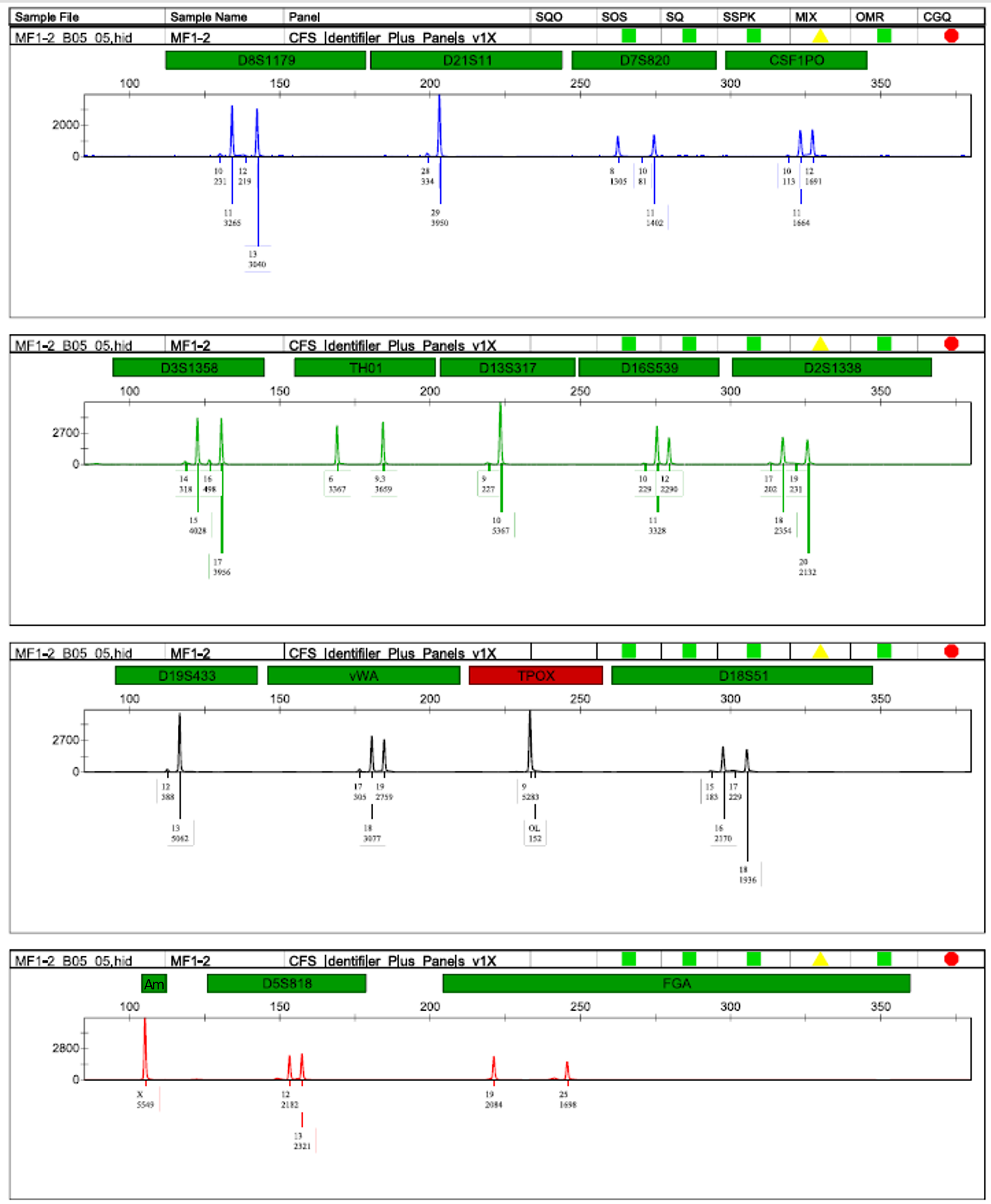


Figure S12. Electrophoretic analysis of NSF for sample 4 generated using standard laboratory analysis techniques.

Data are shown as traces of fluorescence intensity for each channel (blue, green, black, or red) as a function of fragment length (bp). In each panel, labels above the plot (green or red filled boxes) indicate which markers the peaks correspond to, including the sex-typing marker Amelogenin (“Am”) and STR loci D8S1179, D21S11, etc. Labels below the plot (blue, green, black, and red outlined boxes) indicate either the number of repeats for STR loci or X / Y designation for sex typing, as determined using GeneMapper® ID-X (Applied Biosystems), as well as the peak height.

Sample 4 – 1 hr post-coitus – SF, DMF, lab-based DNA analysis, electropherogram


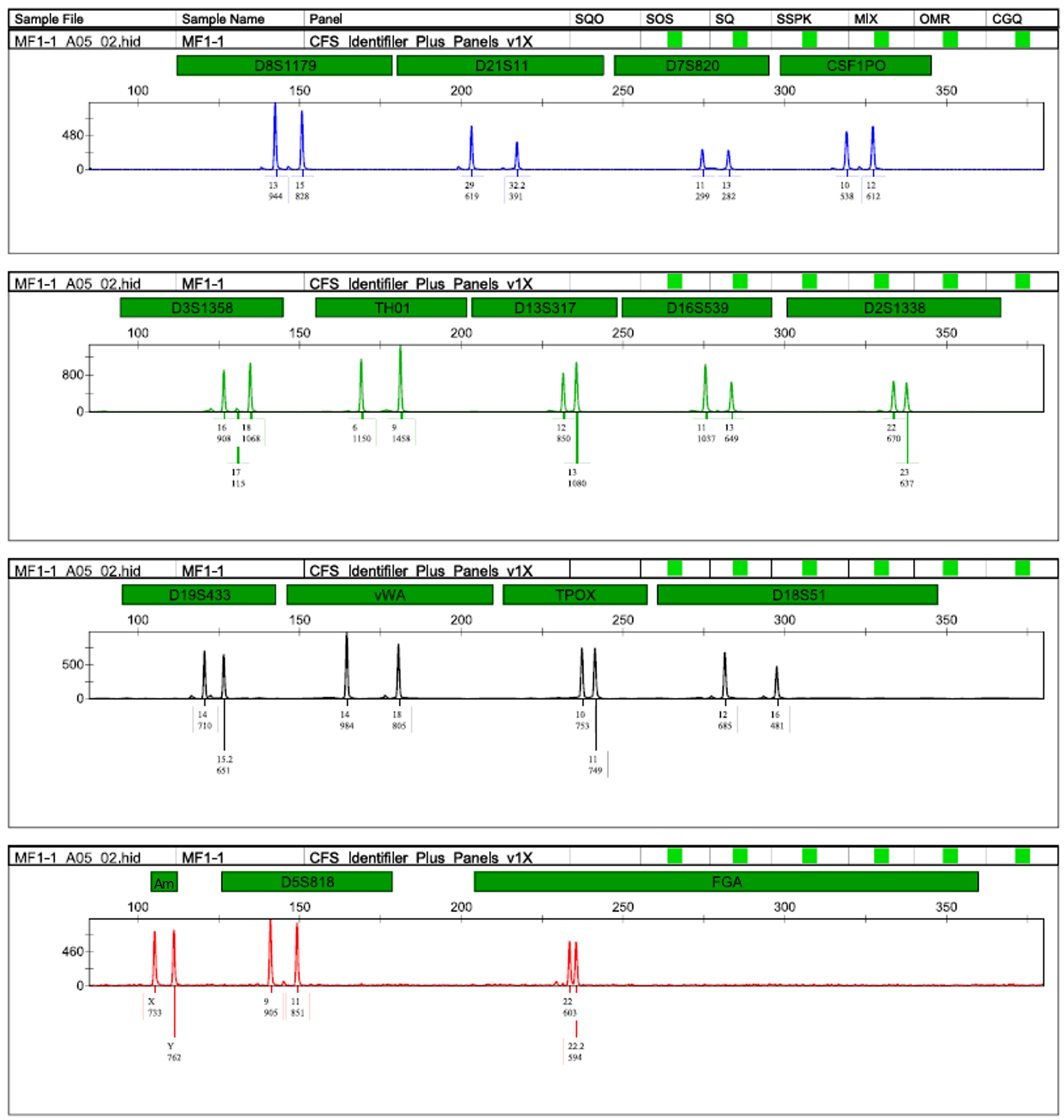


Figure S13. Electrophoretic analysis of SF for sample 4, processed using DMF-DD, generated using standard laboratory analysis techniques.

Data are shown as traces of fluorescence intensity for each channel (blue, green, black, or red) as a function of fragment length (bp). In each panel, labels above the plot (green filled boxes) indicate which markers the peaks correspond to, including the sex-typing marker Amelogenin (“Am”) and STR loci D8S1179, D21S11, etc. Labels below the plot (blue, green, black, and red outlined boxes) indicate either the number of repeats for STR loci or X / Y designation for sex typing, as determined using GeneMapper® ID-X (Applied Biosystems), as well as the peak height.

Sample 4 – 1 hr post-coitus – SF†, DMF, rapid DNA analysis, electropherogram


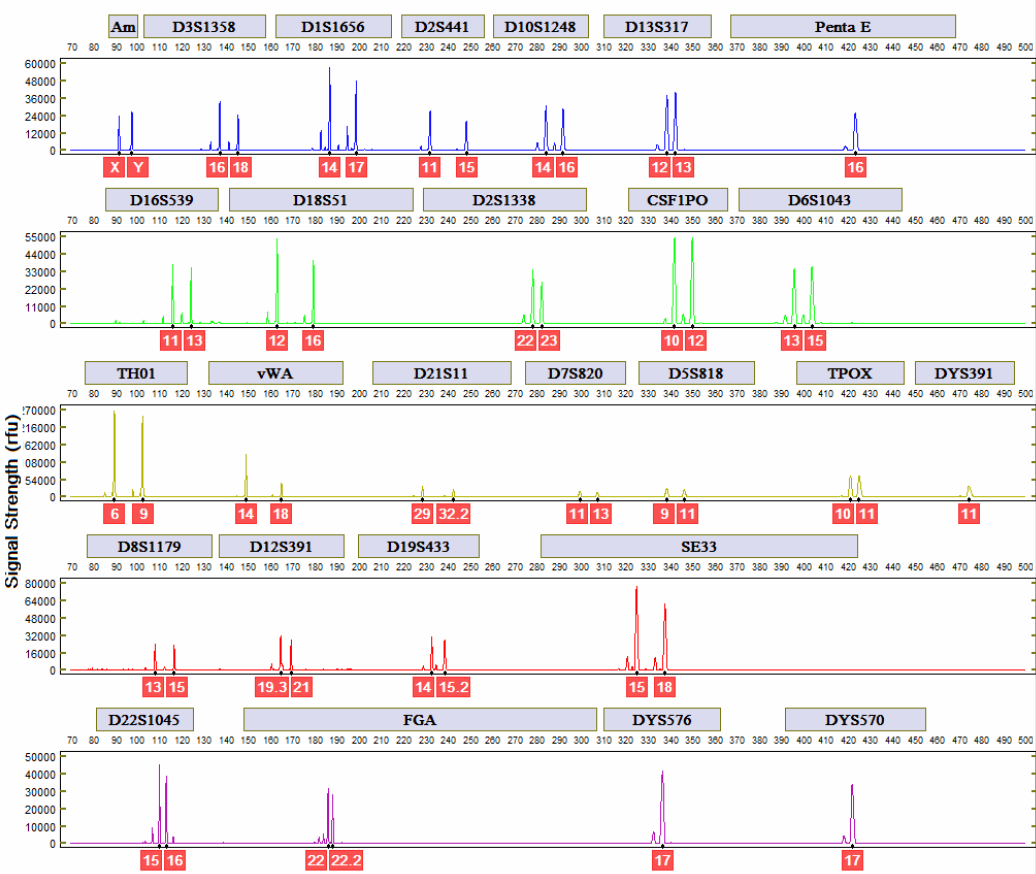


Figure S14. “Rapid DNA” analysis of sample 4.

Data are shown as traces of fluorescence intensity for each label (blue, green, yellow, red, or purple) as a function of fragment length (bp) generated using the ANDE^TM^ 6C system. Labels above the plot (gray filled boxes) indicate which markers the detected peaks correspond to, including the sex-typing marker Amelogenin (“Am”) and STR loci D3S1358, D1S1656, D2S441, D10S1248, D13S317, Penta E, D16S539, D18S51, D2S1338, CSF1P0, D6S1043, TH01, vWA, D21S11, D7S820, D5S818, TPOX, DYS391, D8S1179, D12S391, D19S433, SE33, D22S1045, FGA, DYS576, and DYS570. Labels below the plot (red filled boxes) indicate either the number of repeats for STR loci or X / Y designation for sex typing.

Sample 5 – 3 hrs post-coitus – NSF electropherogram


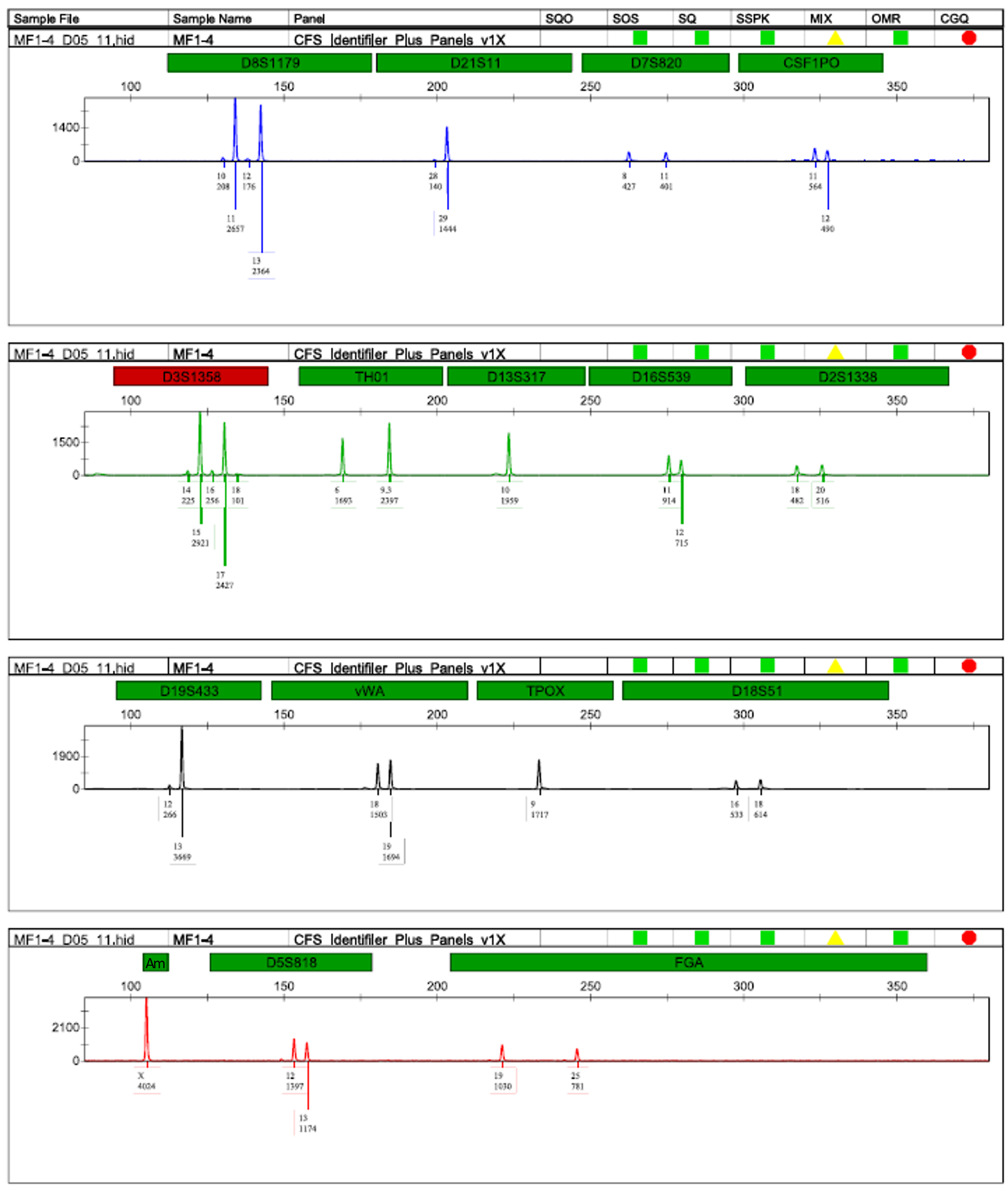


Figure S15. Electrophoretic analysis of NSF for sample 5 generated using standard laboratory analysis techniques.

Data are shown as traces of fluorescence intensity for each channel (blue, green, black, or red) as a function of fragment length (bp). In each panel, labels above the plot (green or red filled boxes) indicate which markers the peaks correspond to, including the sex-typing marker Amelogenin (“Am”) and STR loci D8S1179, D21S11, etc. Labels below the plot (blue, green, black, and red outlined boxes) indicate either the number of repeats for STR loci or X / Y designation for sex typing, as determined using GeneMapper® ID-X (Applied Biosystems), as well as the peak height.

Sample 5 – 3 hrs post-coitus – SF, DMF, lab-based DNA analysis, electropherogram


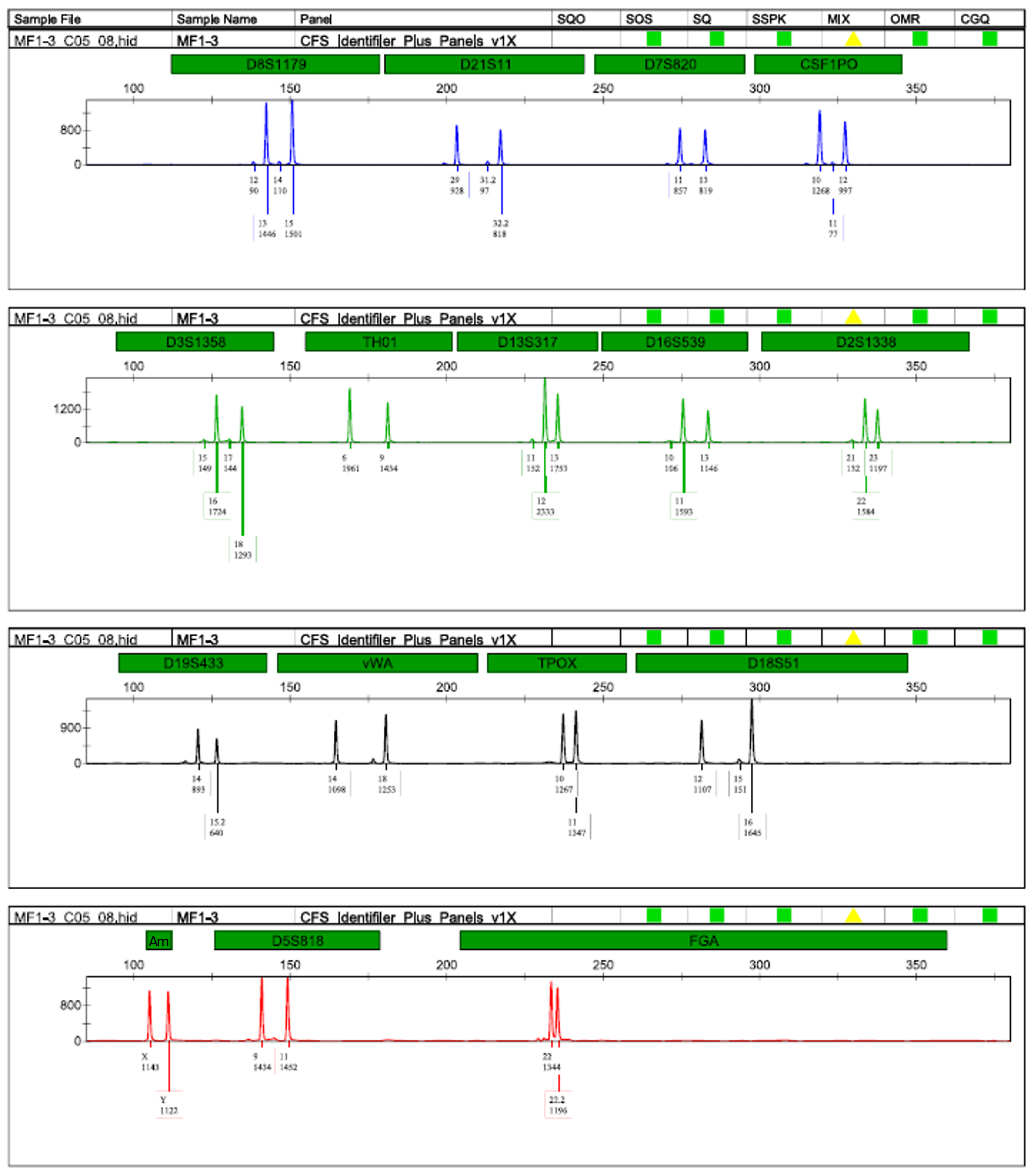


Figure S16. Electrophoretic analysis of SF for sample 5, processed using DMF-DD, generated using standard laboratory analysis techniques.

Data are shown as traces of fluorescence intensity for each channel (blue, green, black, or red) as a function of fragment length (bp). In each panel, labels above the plot (green filled boxes) indicate which markers the peaks correspond to, including the sex-typing marker Amelogenin (“Am”) and STR loci D8S1179, D21S11, etc. Labels below the plot (blue, green, black, and red outlined boxes) indicate either the number of repeats for STR loci or X / Y designation for sex typing, as determined using GeneMapper® ID-X (Applied Biosystems), as well as the peak height.

Sample 5 – 3 hrs post-coitus – SF†, DMF, rapid DNA analysis, electropherogram


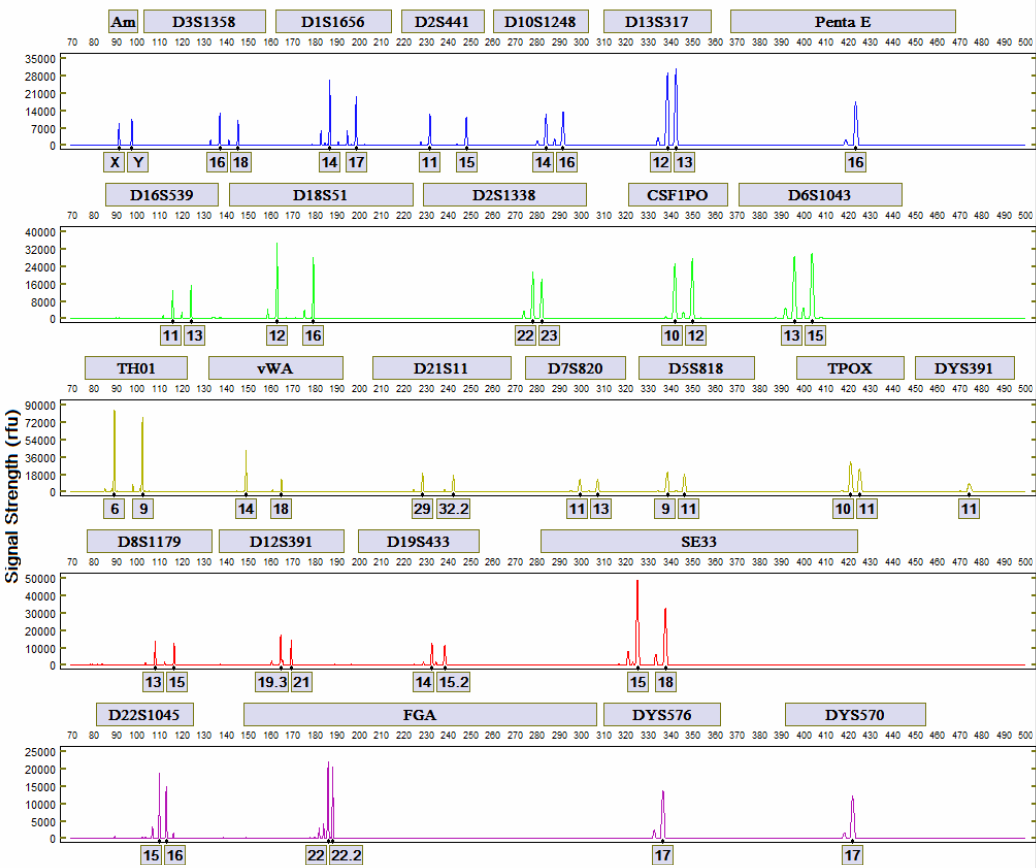


Figure S17. “Rapid DNA” analysis of sample 5.

Data are shown as traces of fluorescence intensity for each label (blue, green, yellow, red, or purple) as a function of fragment length (bp) generated using the ANDE^TM^ 6C system. Labels above the plot (gray filled boxes) indicate which markers the detected peaks correspond to, including the sex-typing marker Amelogenin (“Am”) and STR loci D3S1358, D1S1656, etc. Labels below the plot (grey filled boxes) indicate either the number of repeats for STR loci or X / Y designation for sex typing.

Sample 6 – 6 hrs post-coitus – NSF electropherogram


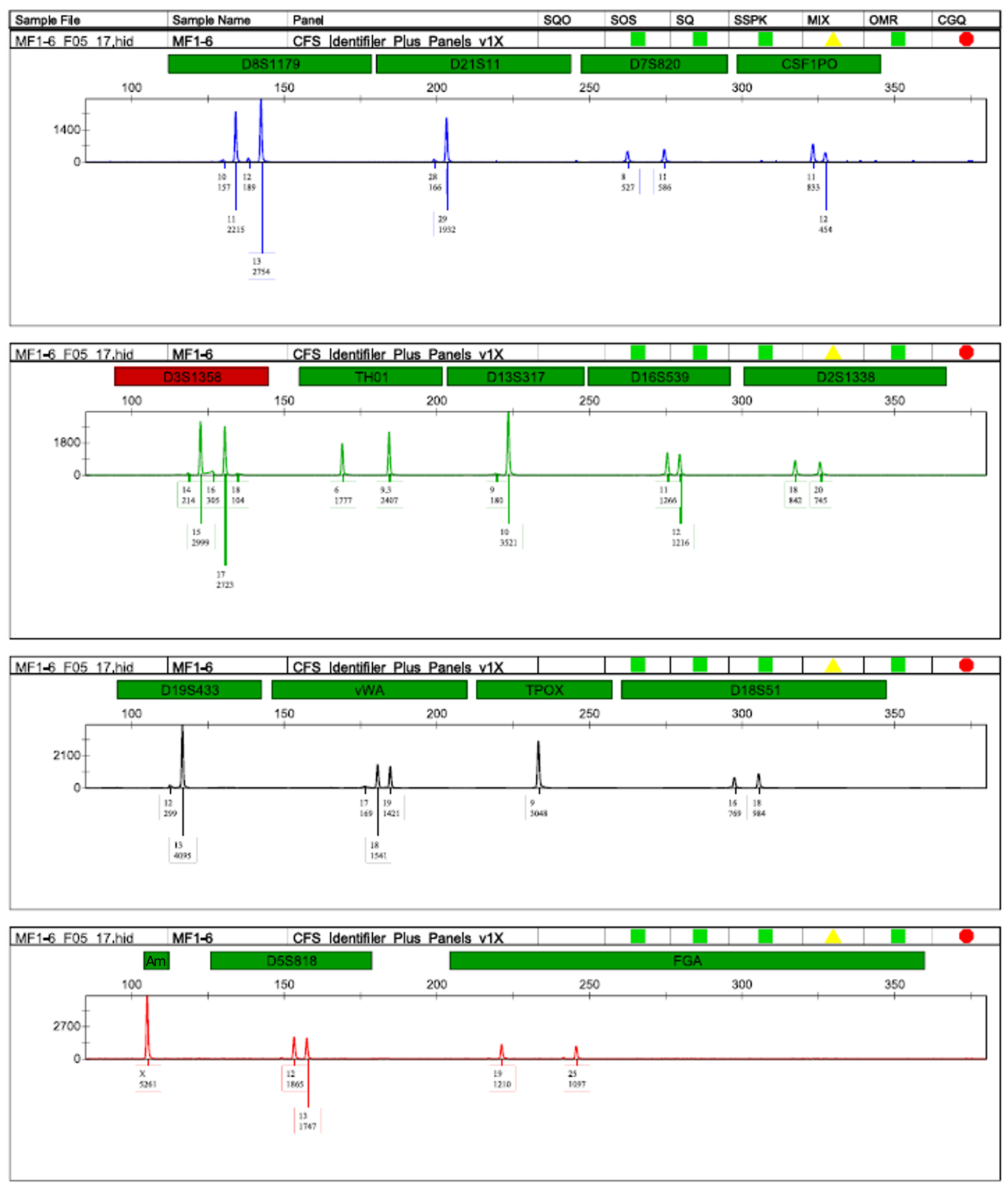


Figure S18. Electrophoretic analysis of NSF for sample 6 generated using standard laboratory analysis techniques.

Data are shown as traces of fluorescence intensity for each channel (blue, green, black, or red) as a function of fragment length (bp). In each panel, labels above the plot (green or red filled boxes) indicate which markers the peaks correspond to, including the sex-typing marker Amelogenin (“Am”) and STR loci D8S1179, D21S11, etc. Labels below the plot (blue, green, black, and red outlined boxes) indicate either the number of repeats for STR loci or X / Y designation for sex typing, as determined using GeneMapper® ID-X (Applied Biosystems), as well as the peak height.

Sample 6 – 6 hrs post-coitus – SF, DMF, lab-based DNA Analysis, electropherogram


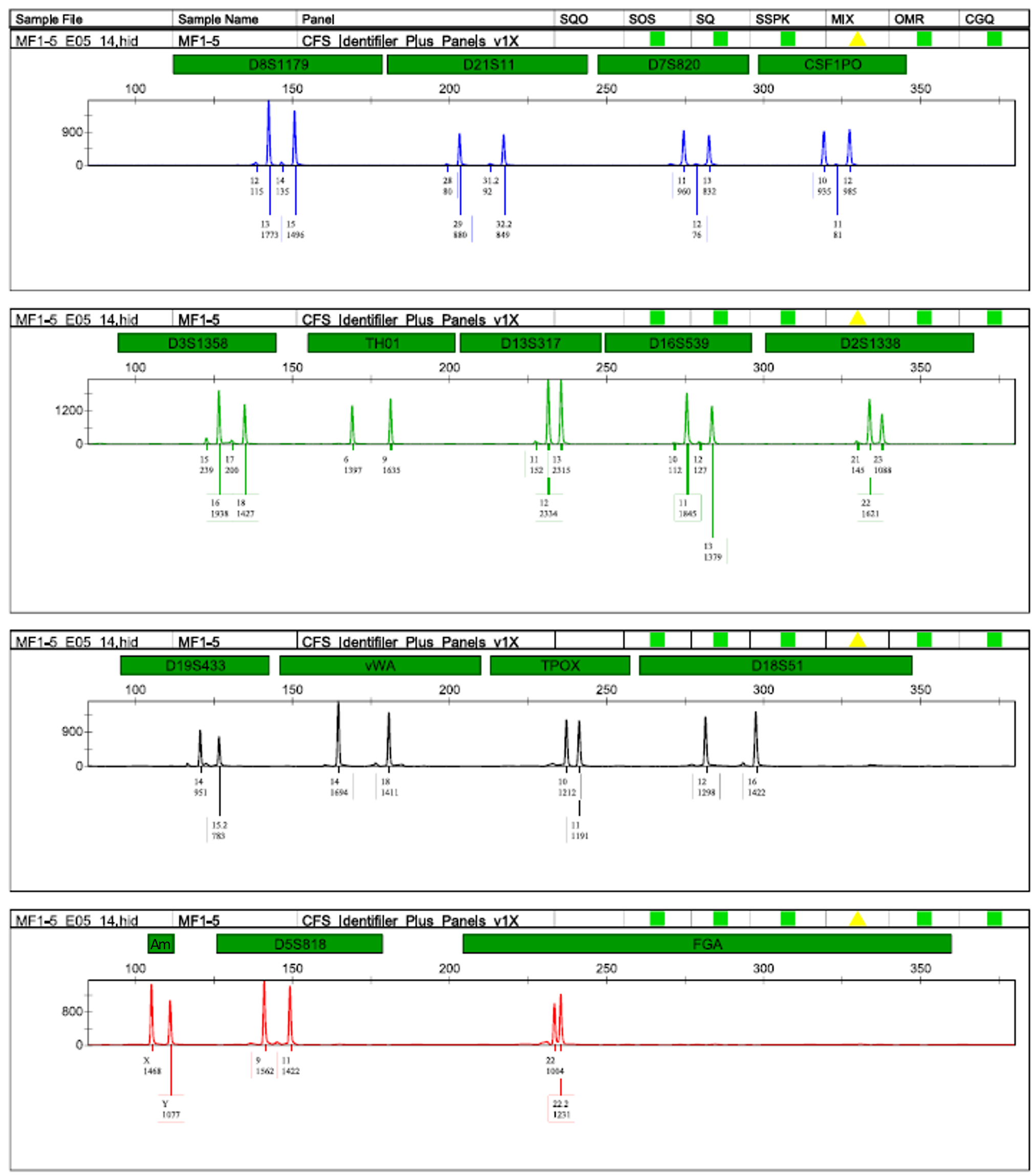


Figure S19. Electrophoretic analysis of SF for sample 6, processed using DMF-DD, generated using standard laboratory analysis techniques.

Data are shown as traces of fluorescence intensity for each channel (blue, green, black, or red) as a function of fragment length (bp). In each panel, labels above the plot (green filled boxes) indicate which markers the peaks correspond to, including the sex-typing marker Amelogenin (“Am”) and STR loci D8S1179, D21S11, etc. Labels below the plot (blue, green, black, and red outlined boxes) indicate either the number of repeats for STR loci or X / Y designation for sex typing, as determined using GeneMapper® ID-X (Applied Biosystems), as well as the peak height.

Sample 6 – 6 hrs post-coitus – SF†, DMF, rapid DNA Analysis, electropherogram


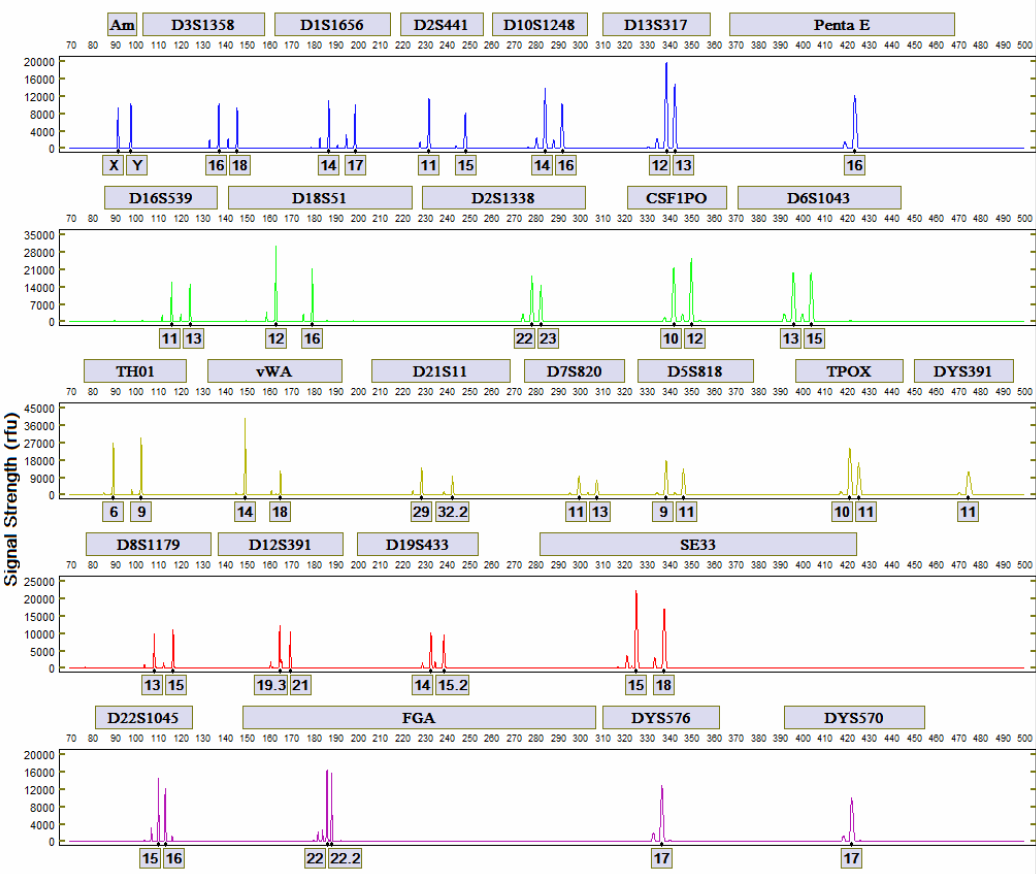


Figure S20. “Rapid DNA” analysis of sample 6.

Data are shown as traces of fluorescence intensity for each label (blue, green, yellow, red, or purple) as a function of fragment length (bp) generated using the ANDE^TM^ 6C system. Labels above the plot (gray filled boxes) indicate which markers the detected peaks correspond to, including the sex-typing marker Amelogenin (“Am”) and STR loci D3S1358, D1S1656, etc. Labels below the plot (grey filled boxes) indicate either the number of repeats for STR loci or X / Y designation for sex typing.

Sample 7 – 12 hrs post-coitus – NSF electropherogram


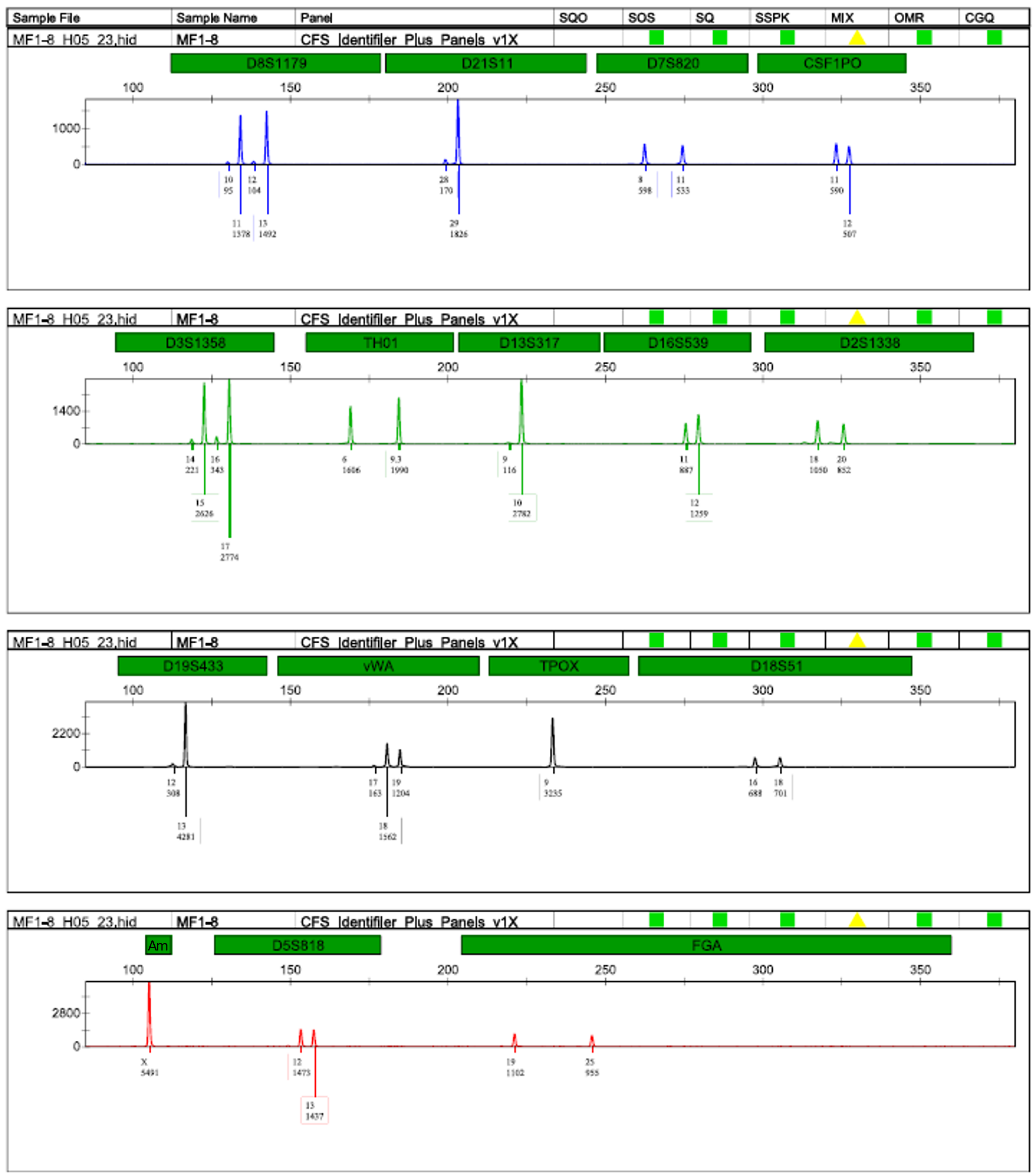


Figure S21. Electrophoretic analysis of NSF for sample 7 generated using standard laboratory analysis techniques.

Data are shown as traces of fluorescence intensity for each channel (blue, green, black, or red) as a function of fragment length (bp). In each panel, labels above the plot (green or red filled boxes) indicate which markers the peaks correspond to, including the sex-typing marker Amelogenin (“Am”) and STR loci D8S1179, D21S11, etc. Labels below the plot (blue, green, black, and red outlined boxes) indicate either the number of repeats for STR loci or X / Y designation for sex typing, as determined using GeneMapper® ID-X (Applied Biosystems), as well as the peak height.

Sample 7 – 12 hrs post-coitus – SF, DMF, lab-based DNA Analysis, electropherogram


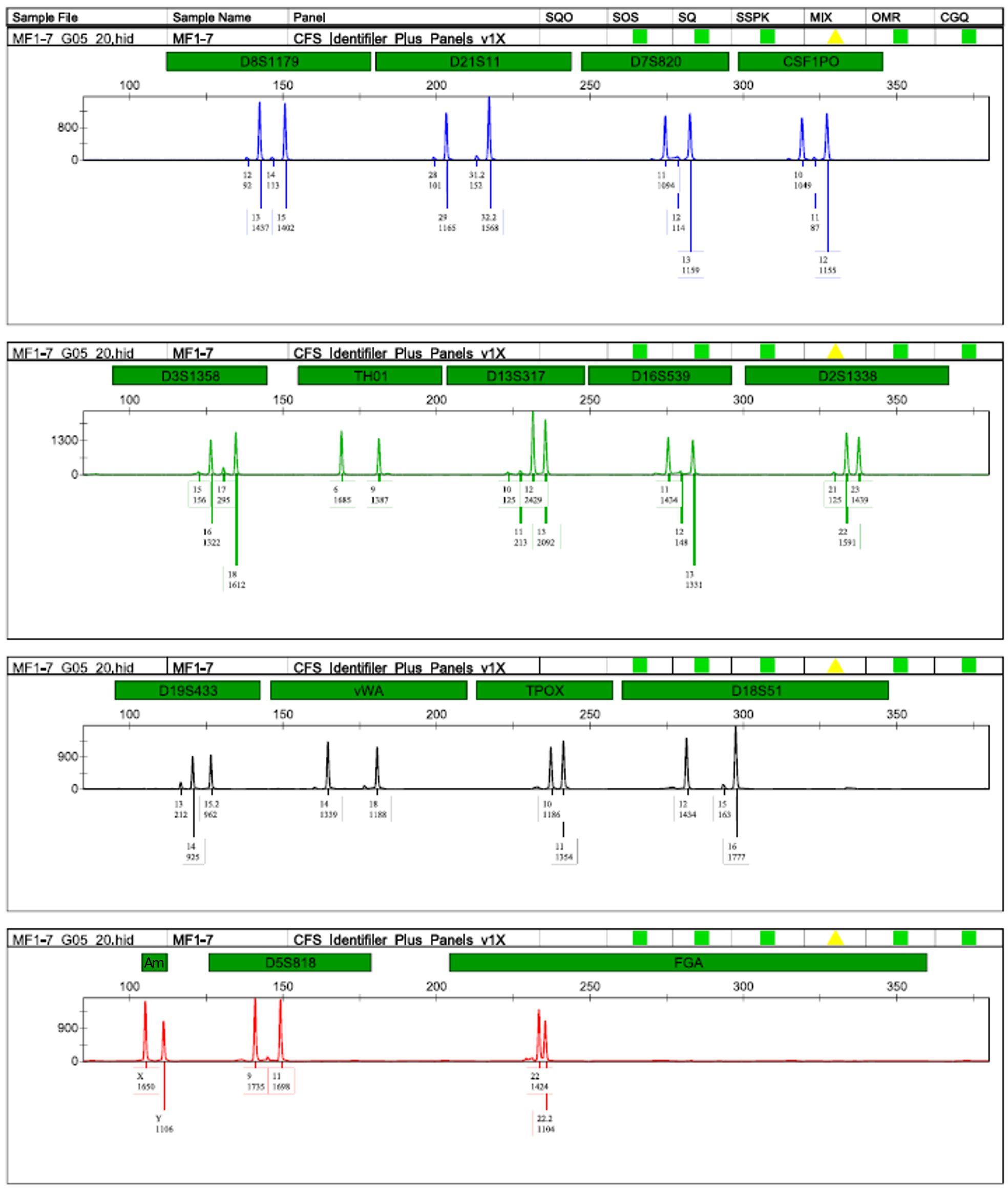


Figure S22. Electrophoretic analysis of SF for sample 7, processed using DMF-DD, generated using standard laboratory analysis techniques.

Data are shown as traces of fluorescence intensity for each channel (blue, green, black, or red) as a function of fragment length (bp). In each panel, labels above the plot (green filled boxes) indicate which markers the peaks correspond to, including the sex-typing marker Amelogenin (“Am”) and STR loci D8S1179, D21S11, etc. Labels below the plot (blue, green, black, and red outlined boxes) indicate either the number of repeats for STR loci or X / Y designation for sex typing, as determined using GeneMapper® ID-X (Applied Biosystems), as well as the peak height.

Sample 7 – 12 hrs post-coitus – SF†, DMF, rapid DNA Analysis, electropherogram


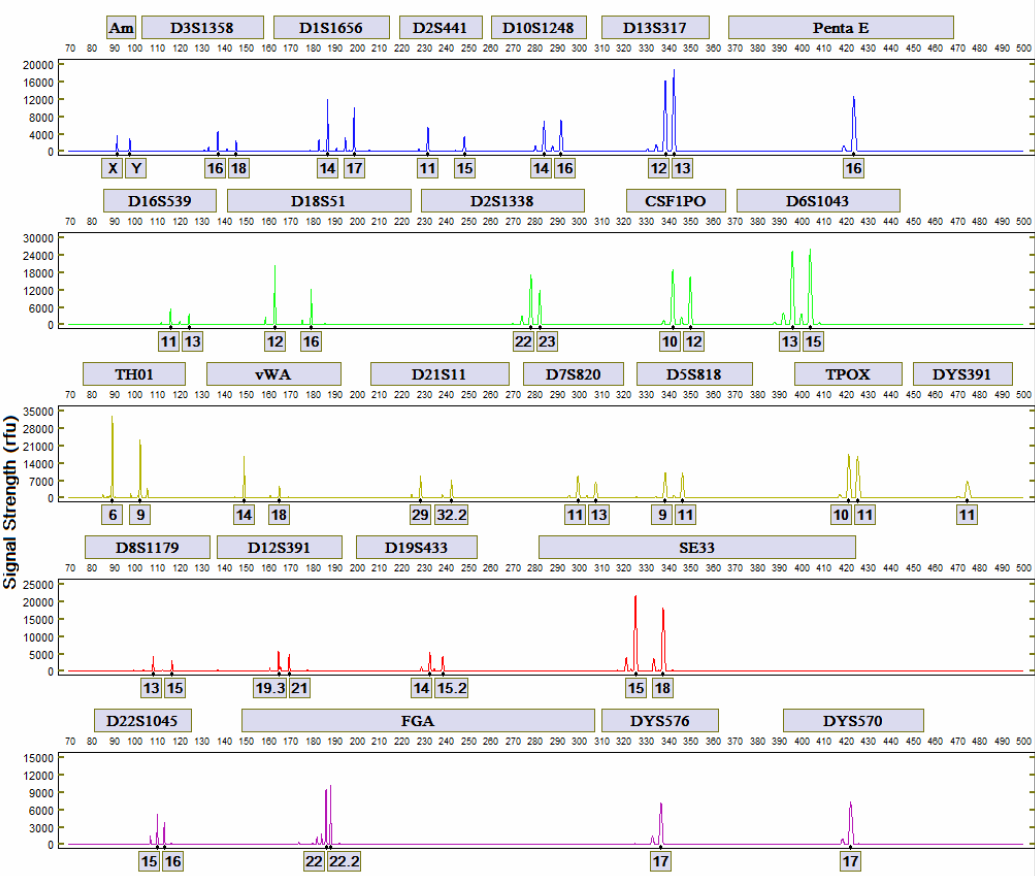


Figure S23. “Rapid DNA” analysis of sample 7.

Data are shown as traces of fluorescence intensity for each label (blue, green, yellow, red, or purple) as a function of fragment length (bp) generated using the ANDE^TM^ 6C system. Labels above the plot (gray filled boxes) indicate which markers the detected peaks correspond to, including the sex-typing marker Amelogenin (“Am”) and STR loci D3S1358, D1S1656, etc. Labels below the plot (grey filled boxes) indicate either the number of repeats for STR loci or X / Y designation for sex typing.

Sample 8 – 24 hrs post-coitus – NSF electropherogram


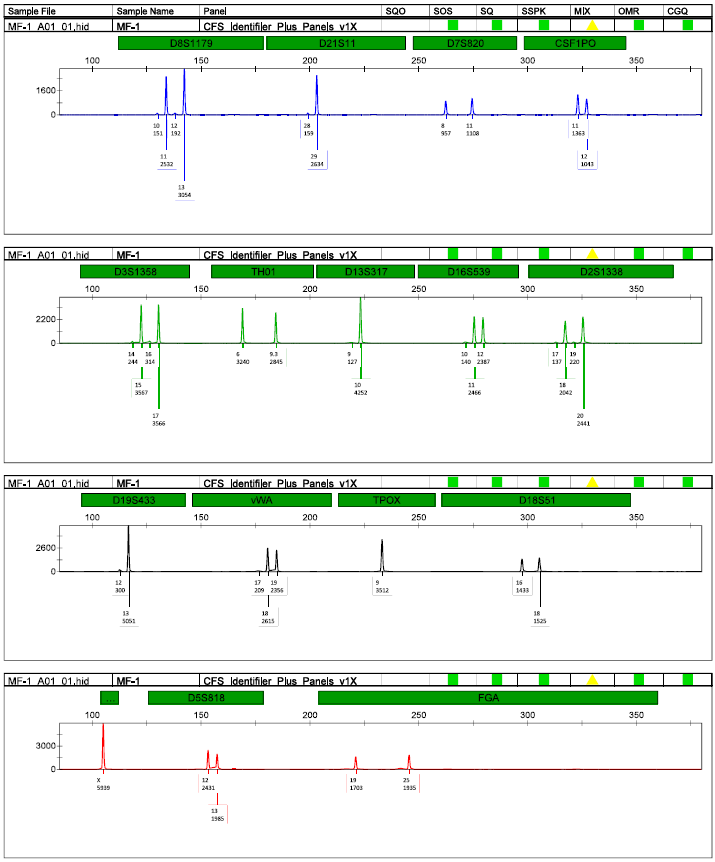


Figure S24. Electrophoretic analysis of NSF for sample 8 generated using standard laboratory analysis techniques.

Data are shown as traces of fluorescence intensity for each channel (blue, green, black, or red) as a function of fragment length (bp). In each panel, labels above the plot (green or red filled boxes) indicate which markers the peaks correspond to, including the sex-typing marker Amelogenin (“Am”) and STR loci D8S1179, D21S11, etc. Labels below the plot (blue, green, black, and red outlined boxes) indicate either the number of repeats for STR loci or X / Y designation for sex typing, as determined using GeneMapper® ID-X (Applied Biosystems), as well as the peak height.

Sample 8 – 24 hrs post-coitus SF, DMF, lab-based DNA Analysis, electropherogram


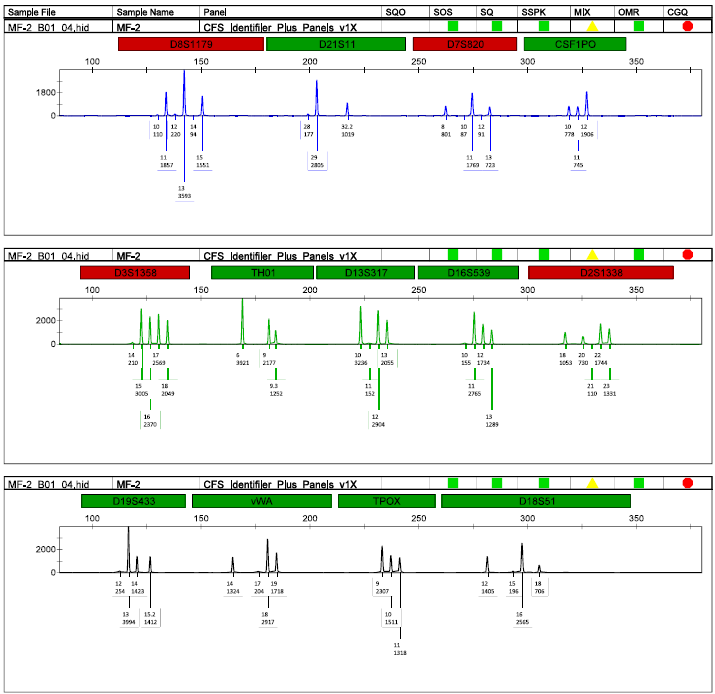


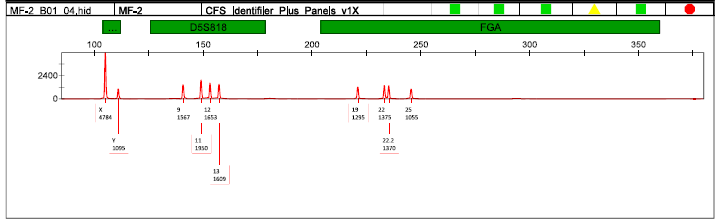


Figure S25. Electrophoretic analysis of SF for sample 8, processed using DMF-DD, generated using standard laboratory analysis techniques.

Data are shown as traces of fluorescence intensity for each channel (blue, green, black, or red) as a function of fragment length (bp). In each panel, labels above the plot (green filled boxes) indicate which markers the peaks correspond to, including the sex-typing marker Amelogenin (“Am”) and STR loci D8S1179, D21S11, etc. Labels below the plot (blue, green, black, and red outlined boxes) indicate either the number of repeats for STR loci or X / Y designation for sex typing, as determined using GeneMapper® ID-X (Applied Biosystems), as well as the peak height.

Sample 8 – 24 hrs post-coitus – SF†, DMF, rapid DNA Analysis, electropherogram


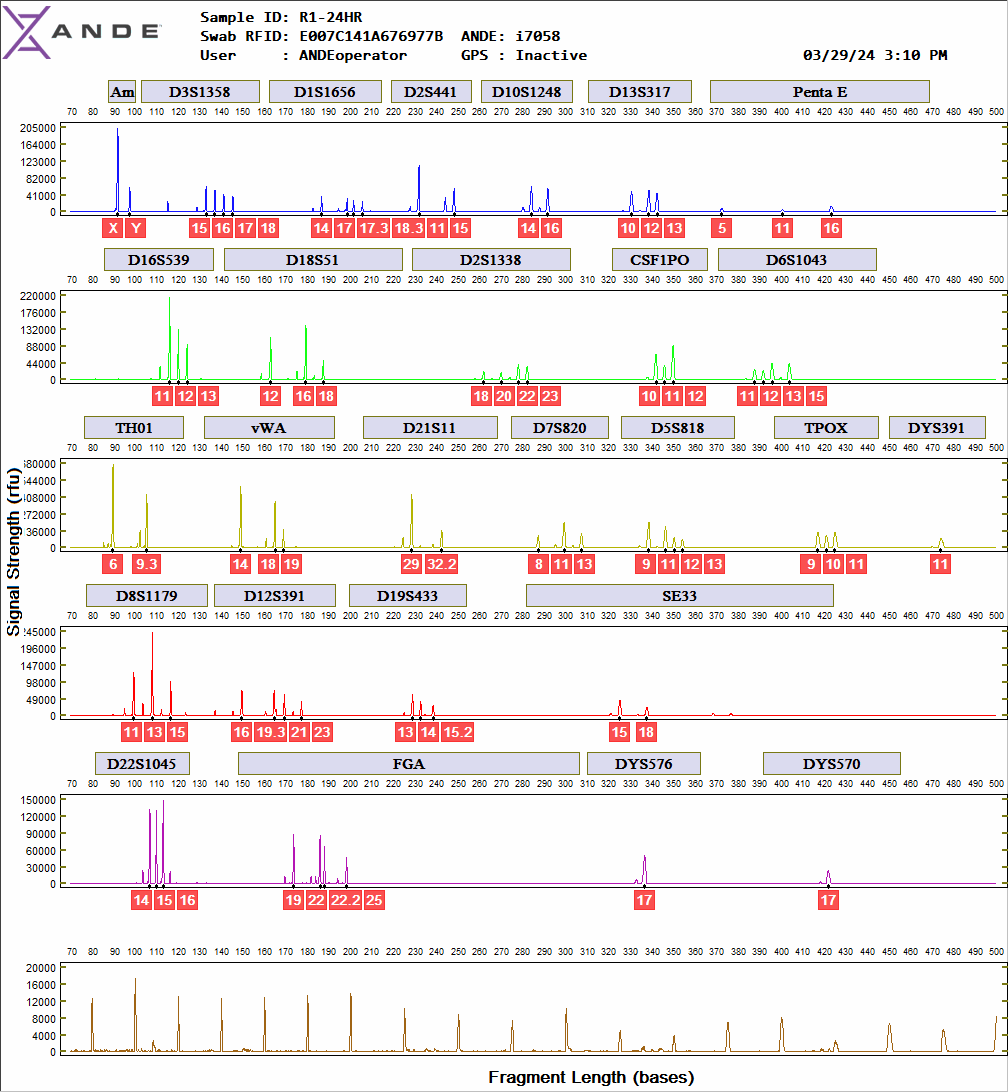


Figure S26. “Rapid DNA” analysis of sample 8.

Data are shown as traces of fluorescence intensity for each label (blue, green, yellow, red, or purple) as a function of fragment length (bp) generated using the ANDE^TM^ 6C system. Labels above the plot (gray filled boxes) indicate which markers the detected peaks correspond to, including the sex-typing marker Amelogenin (“Am”) and STR loci D3S1358, D1S1656, etc. Labels below the plot (red filled boxes) indicate either the number of repeats for STR loci or X / Y designation for sex typing.

Sample 9 – 24 hrs post-coitus – NSF electropherogram


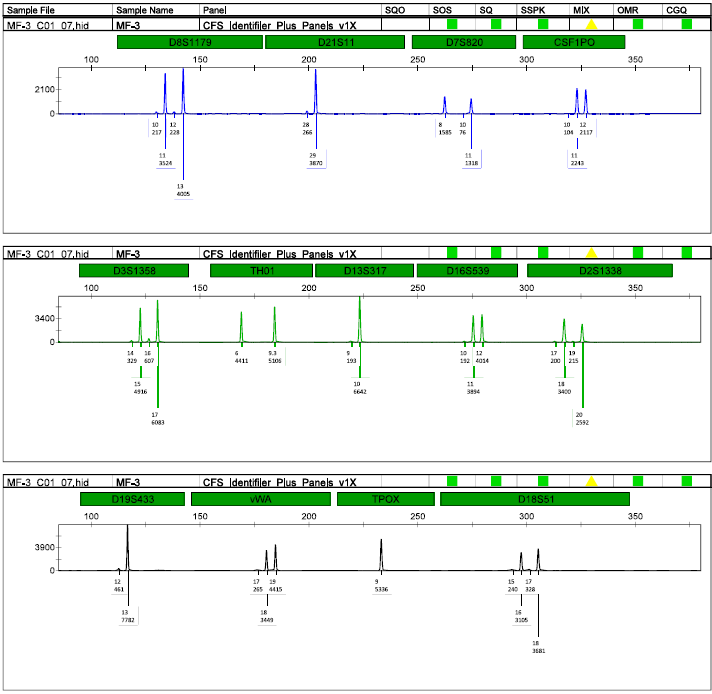


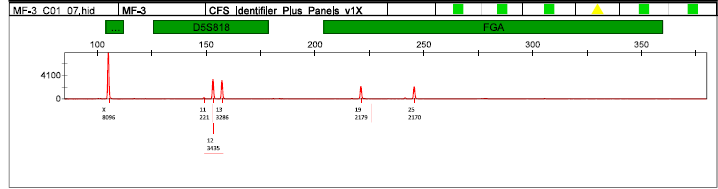


Figure S27. Electrophoretic analysis of NSF for sample 9 generated using standard laboratory analysis techniques.

Data are shown as traces of fluorescence intensity for each channel (blue, green, black, or red) as a function of fragment length (bp). In each panel, labels above the plot (green or red filled boxes) indicate which markers the peaks correspond to, including the sex-typing marker Amelogenin (“Am”) and STR loci D8S1179, D21S11, etc. Labels below the plot (blue, green, black, and red outlined boxes) indicate either the number of repeats for STR loci or X / Y designation for sex typing, as determined using GeneMapper® ID-X (Applied Biosystems), as well as the peak height.

Sample 9 – 24 hrs post-coitus SF, DMF, lab-based DNA Analysis, electropherogram


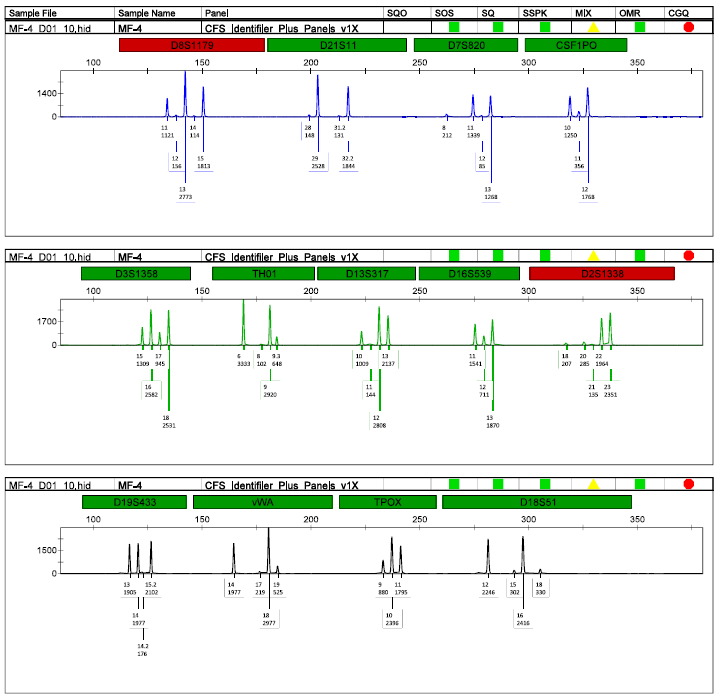


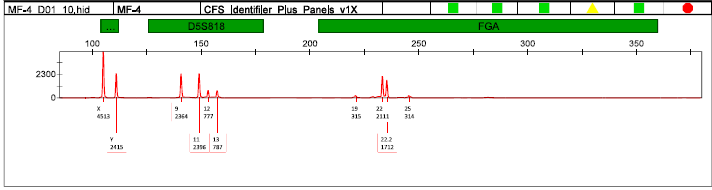


Figure S28. Electrophoretic analysis of SF for sample 9, processed using DMF-DD, generated using standard laboratory analysis techniques.

Data are shown as traces of fluorescence intensity for each channel (blue, green, black, or red) as a function of fragment length (bp). In each panel, labels above the plot (green filled boxes) indicate which markers the peaks correspond to, including the sex-typing marker Amelogenin (“Am”) and STR loci D8S1179, D21S11, etc. Labels below the plot (blue, green, black, and red outlined boxes) indicate either the number of repeats for STR loci or X / Y designation for sex typing, as determined using GeneMapper® ID-X (Applied Biosystems), as well as the peak height.

Sample 9 – 24 hrs post-coitus – SF†, DMF, rapid DNA Analysis, electropherogram


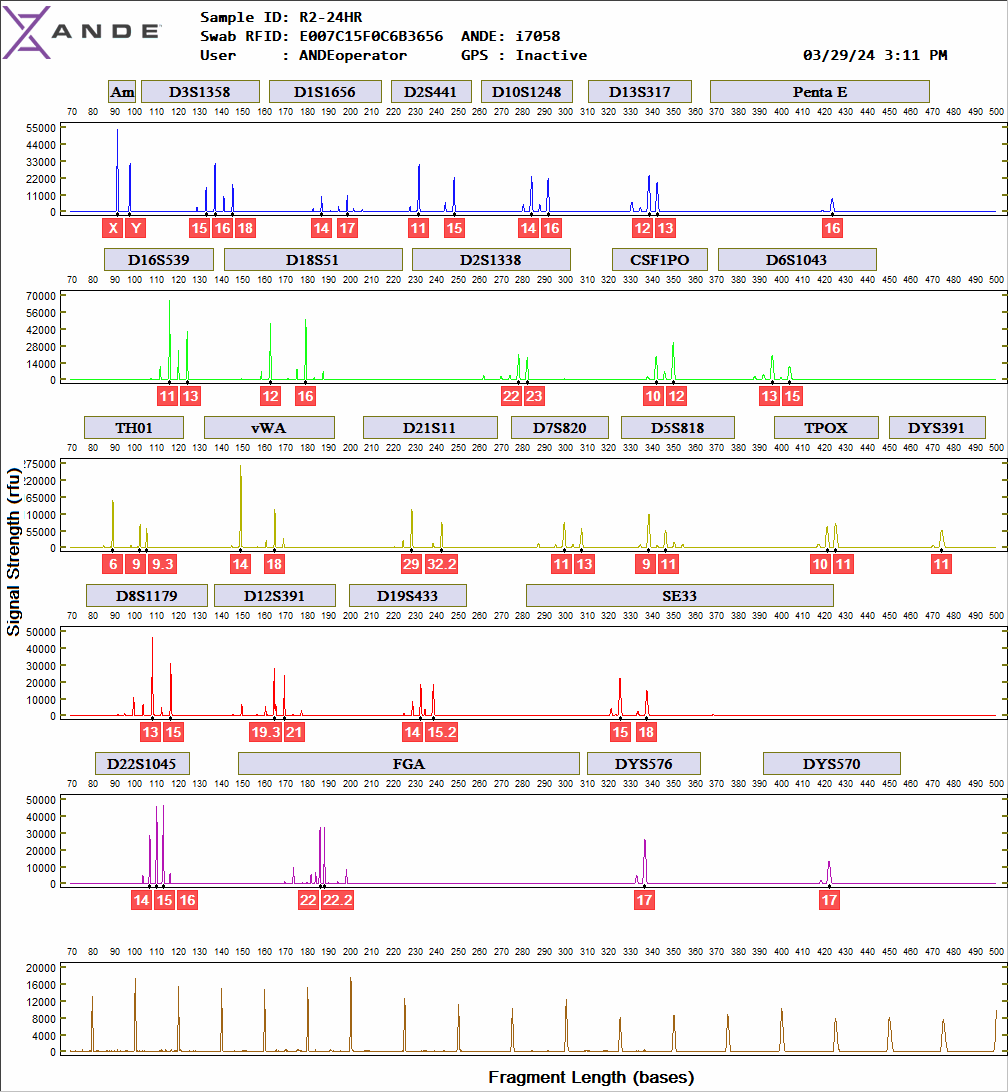


Figure S29. “Rapid DNA” analysis of sample 9.

Data are shown as traces of fluorescence intensity for each label (blue, green, yellow, red, or purple) as a function of fragment length (bp) generated using the ANDE^TM^ 6C system. Labels above the plot (gray filled boxes) indicate which markers the detected peaks correspond to, including the sex-typing marker Amelogenin (“Am”) and STR loci D3S1358, D1S1656, etc. Labels below the plot (red filled boxes) indicate either the number of repeats for STR loci or X / Y designation for sex typing.

Sample 10 – 48 hrs post-coitus – NSF electropherogram


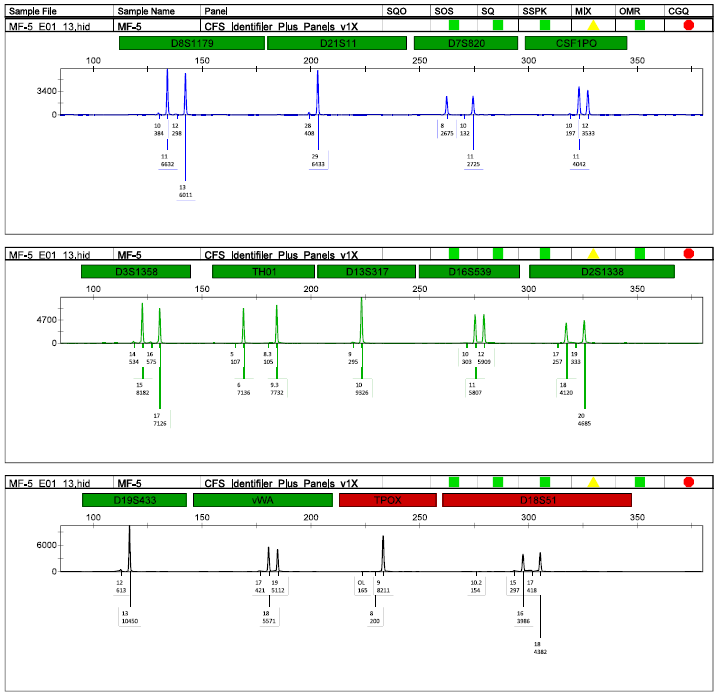


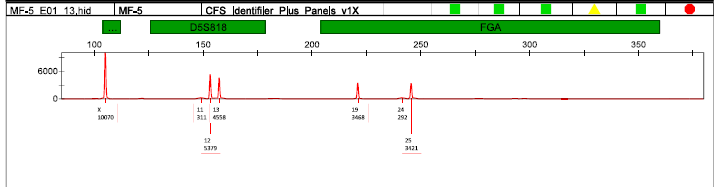


Figure S30. Electrophoretic analysis of NSF for sample 10 generated using standard laboratory analysis techniques.

Data are shown as traces of fluorescence intensity for each channel (blue, green, black, or red) as a function of fragment length (bp). In each panel, labels above the plot (green or red filled boxes) indicate which markers the peaks correspond to, including the sex-typing marker Amelogenin (“Am”) and STR loci D8S1179, D21S11, etc. Labels below the plot (blue, green, black, and red outlined boxes) indicate either the number of repeats for STR loci or X / Y designation for sex typing, as determined using GeneMapper® ID-X (Applied Biosystems), as well as the peak height.

Sample 10 – 48 hrs post-coitus SF, DMF, lab-based DNA Analysis, electropherogram


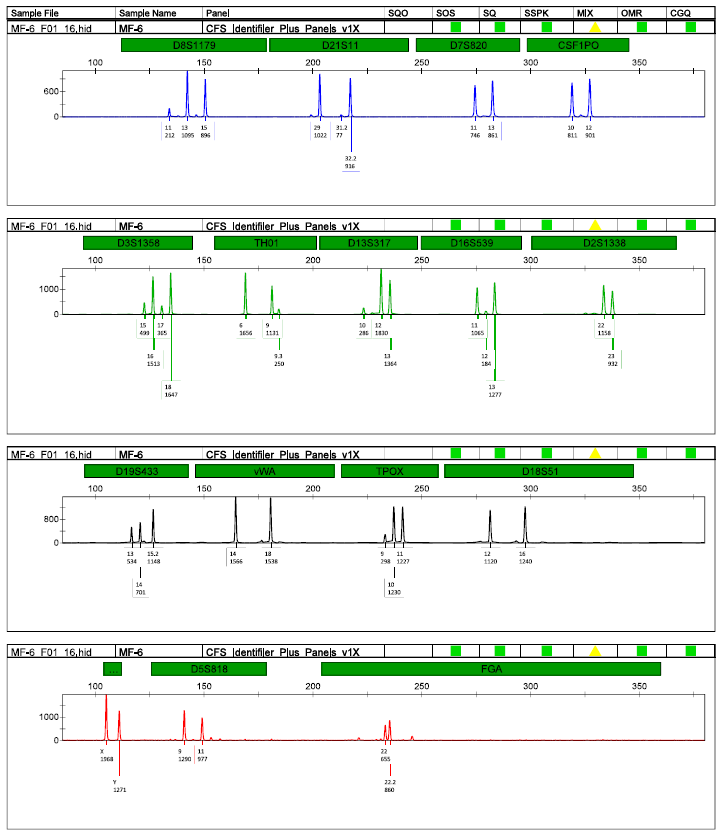


Figure S31. Electrophoretic analysis of SF for sample 10, processed using DMF-DD, generated using standard laboratory analysis techniques.

Data are shown as traces of fluorescence intensity for each channel (blue, green, black, or red) as a function of fragment length (bp). In each panel, labels above the plot (green filled boxes) indicate which markers the peaks correspond to, including the sex-typing marker Amelogenin (“Am”) and STR loci D8S1179, D21S11, etc. Labels below the plot (blue, green, black, and red outlined boxes) indicate either the number of repeats for STR loci or X / Y designation for sex typing, as determined using GeneMapper® ID-X (Applied Biosystems), as well as the peak height.

Sample 10 – 48 hrs post-coitus – SF†, DMF, rapid DNA Analysis, electropherogram


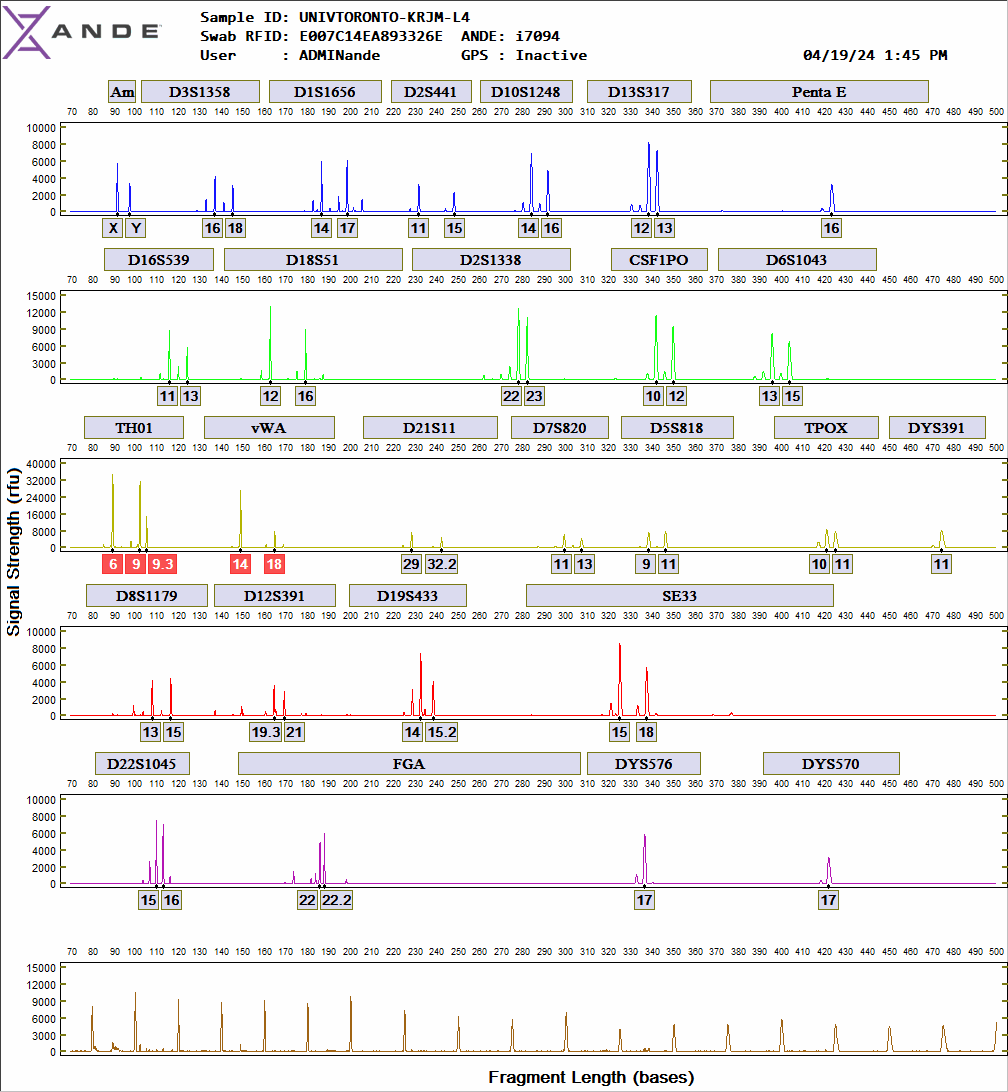


Figure S32. “Rapid DNA” analysis of sample 10.

Data are shown as traces of fluorescence intensity for each label (blue, green, yellow, red, or purple) as a function of fragment length (bp) generated using the ANDE^TM^ 6C system. Labels above the plot (gray filled boxes) indicate which markers the detected peaks correspond to, including the sex-typing marker Amelogenin (“Am”) and STR loci D3S1358, D1S1656, etc. Labels below the plot (grey and red filled boxes) indicate either the number of repeats for STR loci or X / Y designation for sex typing.

Sample 11 – 72 hrs post-coitus – NSF electropherogram


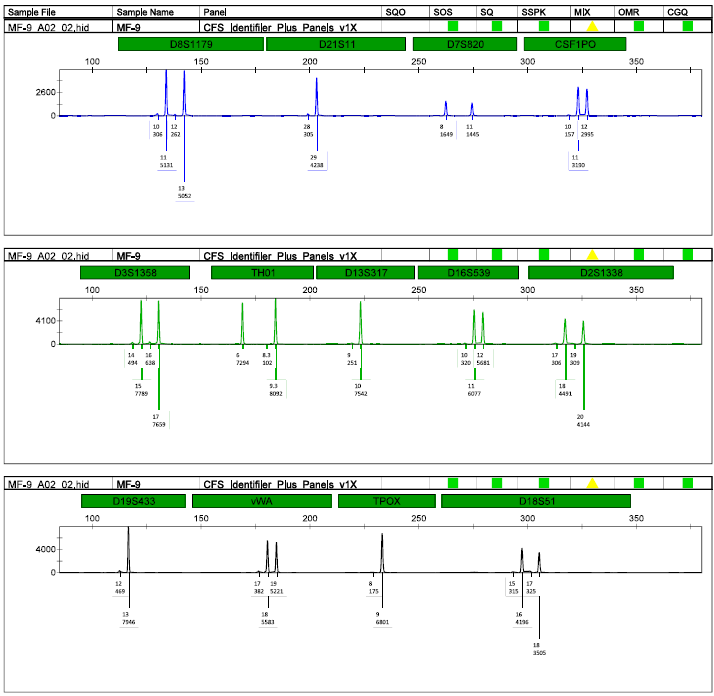


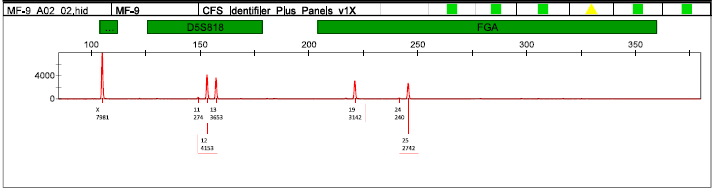


Figure S33. Electrophoretic analysis of NSF for sample 11 generated using standard laboratory analysis techniques.

Data are shown as traces of fluorescence intensity for each channel (blue, green, black, or red) as a function of fragment length (bp). In each panel, labels above the plot (green or red filled boxes) indicate which markers the peaks correspond to, including the sex-typing marker Amelogenin (“Am”) and STR loci D8S1179, D21S11, etc. Labels below the plot (blue, green, black, and red outlined boxes) indicate either the number of repeats for STR loci or X / Y designation for sex typing, as determined using GeneMapper® ID-X (Applied Biosystems), as well as the peak height.

Sample 11 – 72 hrs post-coitus SF, DMF, lab-based DNA Analysis, electropherogram


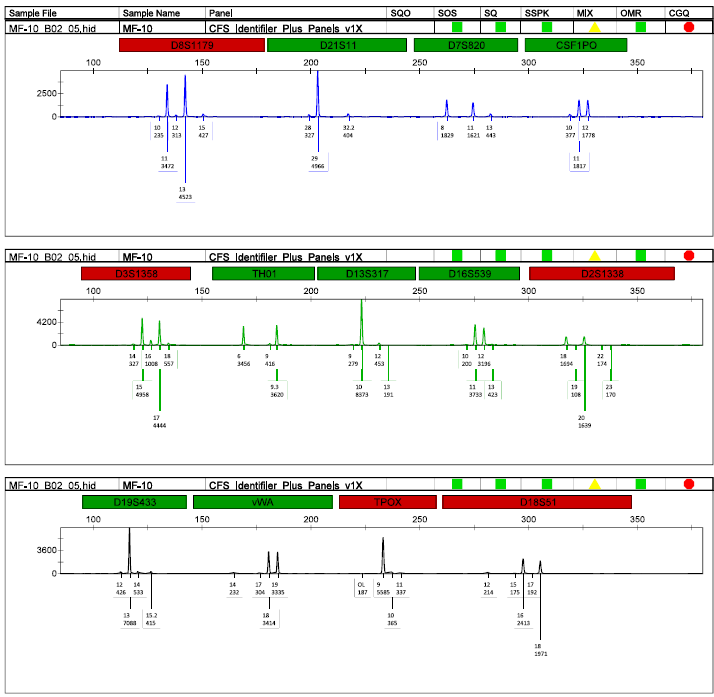


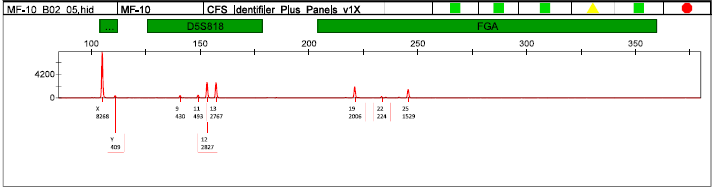


Figure S34. Electrophoretic analysis of SF for sample 11, processed using DMF-DD, generated using standard laboratory analysis techniques.

Data are shown as traces of fluorescence intensity for each channel (blue, green, black, or red) as a function of fragment length (bp). In each panel, labels above the plot (green filled boxes) indicate which markers the peaks correspond to, including the sex-typing marker Amelogenin (“Am”) and STR loci D8S1179, D21S11, etc. Labels below the plot (blue, green, black, and red outlined boxes) indicate either the number of repeats for STR loci or X / Y designation for sex typing, as determined using GeneMapper® ID-X (Applied Biosystems), as well as the peak height.

Sample 11 – 72 hrs post-coitus – SF†, DMF, rapid DNA Analysis, electropherogram


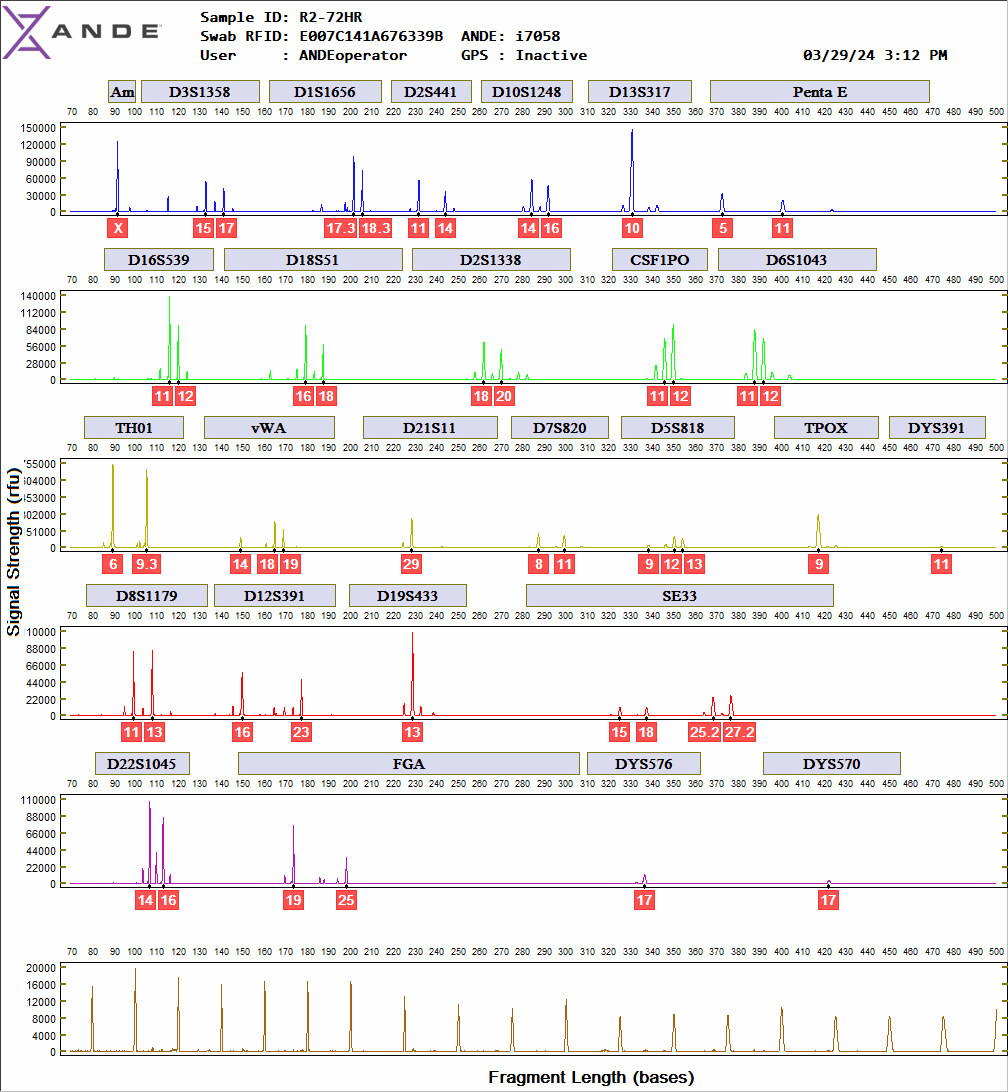


Figure S35. “Rapid DNA” analysis of sample 11.

Data are shown as traces of fluorescence intensity for each label (blue, green, yellow, red, or purple) as a function of fragment length (bp) generated using the ANDE^TM^ 6C system. Labels above the plot (gray filled boxes) indicate which markers the detected peaks correspond to, including the sex-typing marker Amelogenin (“Am”) and STR loci D3S1358, D1S1656, etc. Labels below the plot (red filled boxes) indicate either the number of repeats for STR loci or X / Y designation for sex typing.

**Figure S36. Plot of [*A*]/[*Y*] generated by PCR analysis (PowerQuant®, Promega) for sperm fractions extracted from buccal swabs from a female volunteer spiked with 50 µL semen diluted 1:10.** The conditions evaluated were the original DD method^[8]^ (left), the optimized (“Trial 4”) method implemented by pipettes in tubes (center), and the optimized (“Trial 4”) method implemented by DMF (right). Data are shown as averages ± standard deviation for *n* = 3 technical replicates evaluated for *n* = 3 biological replicates (different swabs) for each condition.

#

# **Supporting References**

[1] M. M. Ewing, J. M. Thompson, R. S. McLaren, V. M. Purpero, K. J. Thomas, P. A. Dobrowski, G. A. Degroot, E. L. Romsos, D. R. Storts, *Forensic Sci. Int. Genet.* **2016**, *23*, 166.

[2] C. T. Sanders, N. Sanchez, J. Ballantyne, D. A. Peterson, *J. Forensic Sci.* **2006**, *51*, 748.

[3] B. E. Krenke, N. Nassif, C. J. Sprecher, C. Knox, M. Schwandt, D. R. Storts, *Forensic Sci. Int. Genet.* **2008**, *3*, 14.

[4] A. Inobeme, V. Nayak, T. J. Mathew, S. Okonkwo, L. Ekwoba, A. I. Ajai, E. Bernard, J. Inobeme, M. Mariam Agbugui, K. R. Singh, *J. Environ. Manage.* **2022**, *309*, 114653.

[5] K. Vanaja, R. H. Shobha Rani, *Clin. Res. Regul. Aff.* **2007**, *24*, 1.

[6] K. Héberger, Chapter 7 - Chemoinformatics—multivariate mathematical–statistical methods for data evaluation in *Med. Appl. Mass Spectrom.* (Eds.: K. Vékey, A. Telekes, A. Vertes), Elsevier, Amsterdam, **2008**, pp. 141–169.

[7] D. C. Carson, D. A. Garvin, K. Gorman, *Automated Processing of Sexual Assault Cases Using Selective Degradation*, **2012**.

[8] H. Wong, J. Mihalovich, *J. Forensic Sci.* **2019**, *64*, 539.
